# Effects of water flow on submerged macrophyte-biofilm systems in constructed wetlands

**DOI:** 10.1038/s41598-018-21080-y

**Published:** 2018-02-08

**Authors:** Bing Han, Songhe Zhang, Peifang Wang, Chao Wang

**Affiliations:** Ministry of Education Key Laboratory of Integrated Regulation and Resource Development on Shallow Lakes, College of Environment, Hohai University, Nanjing, 210098 China

## Abstract

The effects of water flow on the leaf-biofilm interface of *Vallisneria natans* and *Hydrilla verticillata* were investigated using artificial plants as the control. Water flow inhibited the growth of two species of submerged macrophytes, reduced oxygen concentrations in plant leaves and changed oxygen profiles at the leaf-biofilm interface. The results from confocal laser scanning microscopy and multifractal analysis showed that water flow reduced biofilm thickness, changed biofilm topographic characterization and increased the percentages of single colony-like biofilm patches. A cluster analysis revealed that the bacterial compositions in biofilms were determined mainly by substrate types and were different from those in sediments. However, water flow increased the bacterial diversity in biofilms in terms of operational taxonomic unit numbers and Shannon Indices. Our results indicated that water flow can be used to regulate the biomass, distribution and bacterial diversities of epiphytic biofilms in constructed wetlands dominated by submerged macrophytes.

## Introduction

Submerged macrophytes growing under water surface play an important role in water environments and in constructed wetlands (CWs). They are able to take up nutrients from both the sediment and the surrounding water column^1^, and they show advantages in reducing water turbidity caused by phytoplankton and suspended solids^2^. Moreover, submerged macrophytes can provide oxygen, food and shelter for other organisms in the surrounding water column^3–5^. Furthermore, due to the submerged lifestyle, submerged macrophytes are natural substrates for microorganisms (known as biofilm or epiphytic microbes)^6^, which have been universally recognized as a critical factor in the wetland N removal process due to the nitrification and denitrification of bacteria and archaea^7^. Though the role of submerged macrophytes has attracted the attention of researchers in recent years, little is known about the submerged macrophyte-biofilm systems in aquatic ecosystems.

There are complex interactions between submerged macrophytes and biofilms. Submerged macrophytes can expand the niche of epiphytic biofilms by individual growth and expansion^8^. Submerged macrophytes might provide nutrients as well as allelopathic substances to epiphytic microbes, leading to diverse and host specific epiphytic bacterial communities^7,9^. Besides, the growth status of submerged macrophytes showed greater effect on epiphytic bacterial community than the seasonal variation of environmental conditions^10^. During the daytime, submerged macrophytes provide oxygen for nitrifiers in epiphytic biofilms^6^. At nighttime, respiratory consumption in the dense stands of submerged vegetation regions might result in a shift from aerobic to anaerobic bacterial respiration, which is beneficial for denitrification^6^. However, excess biofilms could do harm to the growth of submerged macrophytes. The decline of growth and diversities of submerged macrophyte communities in aquatic ecosystems have been ascribed to the excessive growth of biofilm microbes (including algae), which would attenuate the available nutrients and light to submerged plants^11,12^. The dilemma was usually stimulated by high nutrient loading in the eutrophic waters^13^. Therefore, it is important to find a way to balance the growth of biofilms and submerged macrophytes (e.g. inhibiting the excess biofilm, optimizing mass transfer, improving the richness of biofilm bacterial and archaea) to maintain a stable performance in CWs.

Hydraulic forces generated by waves and flow strongly influence the growth, distribution and species diversity of submerged macrophytes in aquatic environments^14^. Meanwhile, hydraulic conditions modulate the bacterial density, community structure, spatial distribution, and activities (respiration, potential nitrification, denitrification and methanogenesis) of the microbes through adjusting the attachment and detachment of bacteria, the distribution of extracellular polymeric substances and the mass transfer at the biofilm-water interface^15–17^. In addition, water flow can cause the suspension of sediments and carry particles (including microbes) to the surface of submerged plants^18^. Flow rate is an important design parameter of CWs, especially in the submerged macrophyte vegetation zone^19^. To date, studies of the effect of water flow on biofilms have been focused on abiotic substrates (e.g., glass slides, cobbles, riverbeds and pipes in drinking water distribution systems)^15,20–23^, and little is known about how the water flow influences epiphytic biofilms on submerged macrophytes.

Unlike on rigid non-living materials, the surface properties of submerged macrophytes are flexible and animate. The flexibility reduces the physical damage of water flow to the submerged macrophytes by decreasing the water flow and shear force at the leaf-water interface to different extent depending on the degree of architecture complexity of submerged macrophytes^14,24^. The animate host would be growing and interacting with epiphytic biofilms throughout its lifetime through physiological activities (e.g. individual growth^8^, phyllosphere oxygen profiles^6^, allelopathic compounds secretion^9^). Both of these factors make the situation of epiphytic microbes more complex than their counterparts on rigid non-living materials. A recent report showed that there were significant differences in bacterial communities between samples from sediments and biofilm in aquatic environment and between samples from vegetated sediments and unvegetated sediments^25^. However, no studies have systematically investigated the effects of water flow on the biofilms attached to the surface of submerged macrophytes.

Therefore, except the shear force, the epiphytic biofilms might also be affected by the hosts and water flow-driven suspended sediment including microbes, which can be absorbed by/attach to plant surface. In the present study, two species of submerged macrophytes with different morphological features and one type of artificial plant (control) were planted in an experimental setup (Supplementary Fig. 1) with a flume (water flow) and a tank (static water) to fill the gap in knowledge about the effects of water flow on submerged macrophyte-biofilm systems by 1) exploring the effects of water flow on the growth of the submerged macrophytes and the oxygen profiles around and/or in the plant leaf-biofilm systems, 2) investigating the response of biofilms on submerged macrophytes in terms of microbe density and distribution, and 3) comparing the bacterial communities in biofilms on macrophytes with those in vegetated sediments and unvegetated sediments in the experimental setup.

## Results

### Plant growth rates and microbial densities in biofilm

In this study, two species of submerged plants *V. natans* and *H. verticillata* (artificial plants used as control) were employed to analyze the response of biofilm-leaves to water flow in a tank-flume cycling system (Supplementary Fig. 1). Leaves of *V. natans* have rounded tips, and definite raised veins and arise in clusters from their roots, while leaves of *H. verticillata* are arranged in whorls around the stem (Supplementary Fig. 2). The impacts of two species of plants on water flow were monitored and plant growth altered the vertical distribution of water velocity in the plant zone (Supplementary Fig. 3). Though these plants have different morphological traits, they showed similar effects on the vertical distribution of water velocity (Supplementary Fig. 3).

**Figure 1:**
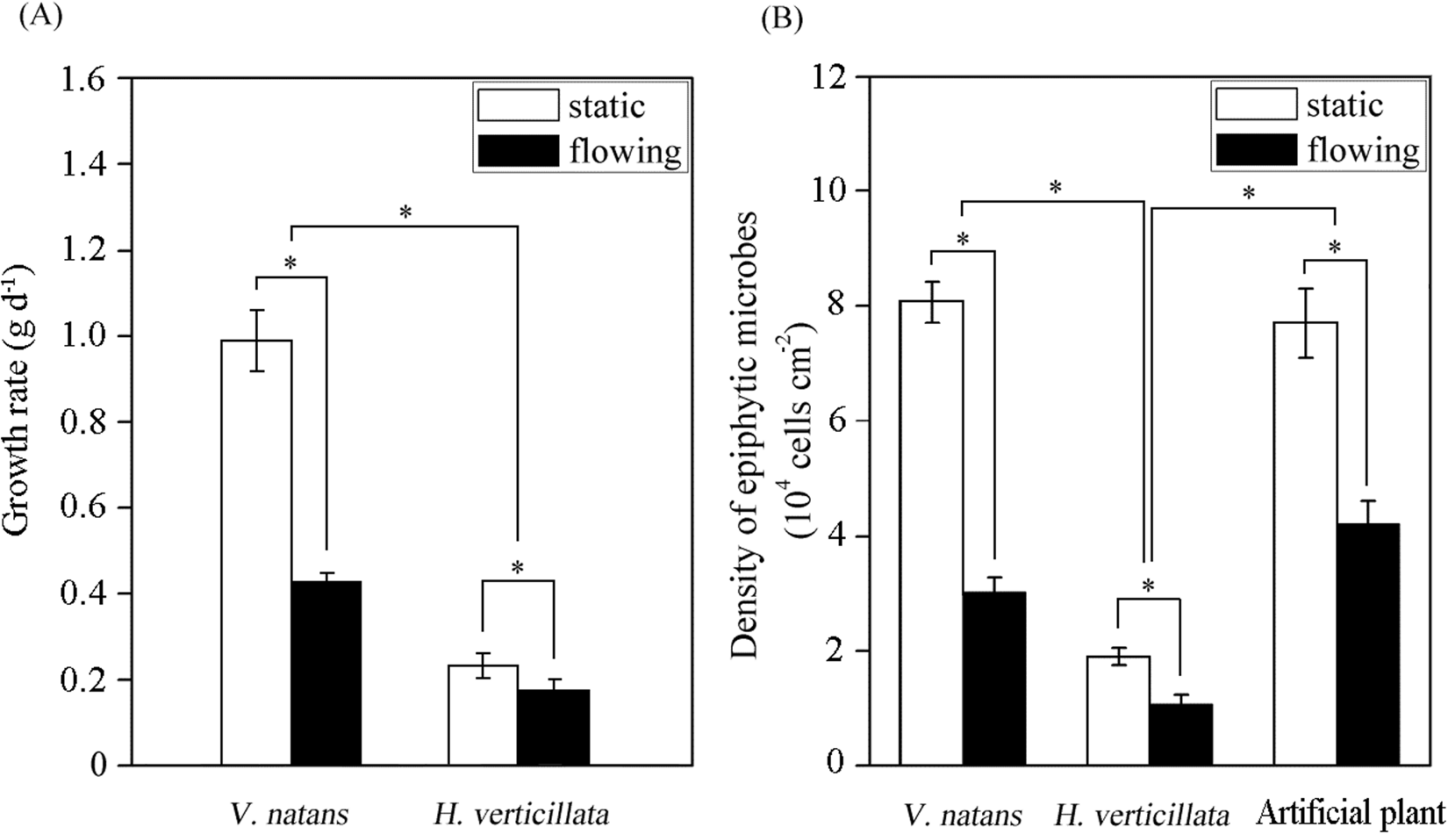
Response of growth rates (**A**, *n* = 6) and epiphytic microbe densities (**B**, *n* = 3) to water flow. *Indicates a significant difference between two groups according to two-way ANOVA (*p* < 0.05).

In the static water, the growth rates of *V. natans* and *H. verticillata* were 0.99 ± 0.071 and 0.23 ± 0.029 g d^−1^ plant^−1^, respectively, whereas in the flowing water, the values were 0.43 ± 0.022 and 0.18 ± 0.023 g d^−1^ plant^−1^, respectively (Fig. 1A). The two-way ANOVA analysis showed that both flow treatment and host species influenced the growth rates significantly (*F*_flow treatment × species_ = 222.0, *p* < 0.001; *F*_flow treatment_ = 334.7, *p* < 0.001; and *F*_species_ = 888.1, *p* < 0.001). These results suggested that water flow decreased the growth rates of the two species of submerged macrophytes, and the growth rate of *V. natans* was larger than that of *H. verticillata*.

Artificial plants were used as a control to monitor the effects of water flow on biofilm formation. The microbial densities on the leaves of artificial plants, *V. natans* and *H. verticillata* were 7.7 × 10^4^ ± 6009 cells cm^−2^, 8.1 × 10^4^ ± 3522 cells cm^−2^ and 1.9 × 10^4^ ± 1542 cells cm^−2^ in the static water, respectively, and were 4.2 × 10^4^ ± 4022 cells cm^−2^, 3.0 × 10^4^ ± 2699 cells cm^−2^ and 1.1 × 10^4^ ± 1674 cells cm^−2^, respectively, in the flowing water (Fig. 1B). The two-way ANOVA analysis on the data of microbial density showed that values of *F*_flow treatment × species_, *F*_flow treatment_ and *F*_species_ were 53.4, 341.7 and 284.8 (*p* < 0.001), respectively. The results revealed that both flow treatment and host species significantly affected the densities of epiphytic microbes on the leaf surfaces of the plants. Water flow decreased the densities of epiphytic microbes on the leaf surface of the plants in the 21 days. No significant difference (*p* = 0.154) was found between data of microbial densities from artificial plants and *V. natans*, whereas data from artificial plants/*V. natans* were larger than those from *H. verticillata* (*p* < 0.001).

### Phyllosphere oxygen profiles

**Figure 2:**
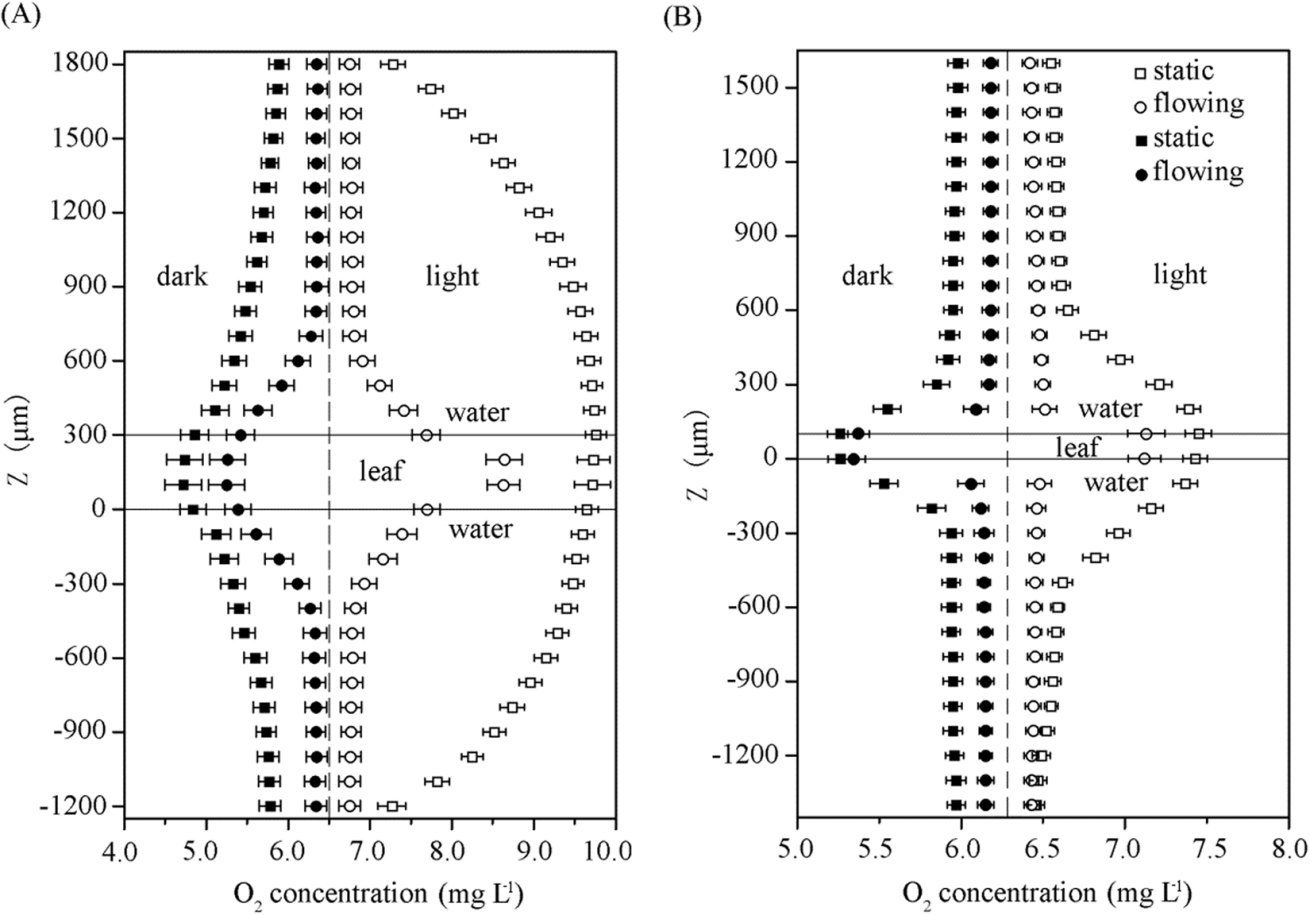
Oxygen profiles (*n* = 6) measured at the phyllosphere of *V. natans* (**A**) and *H. verticillata* (**B**). The vertical axis (Z) showed the range of the puncture test and the platform of the object stage was set to be zero plane.

A puncture test was performed to detect the oxygen profiles in the phyllosphere of two species of submerged macrophytes using a microelectrode system. In general, the oxygen concentrations in plant leaves were higher than those in the water column during the daytime, but the reverse trend was detected at night (Fig. 2). For the two species of submerged macrophytes, sharper gradient of oxygen concentrations was found at the phyllosphere oxygen profiles in the flowing water. For *V. natans*, the rates of oxygen change with depth under light were 1.55 ± 0.096 mg L^−1^ mm^−1^ in the static water and 3.48 ± 0.302 mg L^−1^ mm^−1^ in the flowing water, respectively, with the rates in the dark −0.77 ± 0.073 mg L^−1^ mm^−1^ in the static water and −1.65 ± 0.041 mg L^−1^ mm^−1^ in the flowing water. The change rates of *H. verticillata* under light were 1.58 ± 0.067 mg L^−1^ mm^−1^ in the static water and 6.35 ± 0.723 mg L^−1^ mm^−1^ in the flowing water, respectively, whereas the change rates of *H. verticillata* in the dark were −2.07 ± 0.171 mg L^−1^ mm^−1^ in the static water and −3.70 ± 0.302 mg L^−1^ mm^−1^ in the flowing water, respectively. The two-way ANOVA analysis showed that both flow treatment and host species influenced the average slope of the phyllosphere oxygen profiles significantly during the daytime (*F*_flow treatment × species_ = 77.3, *p* < 0.001; *F*_flow treatment_ = 429.0, *p* < 0.001; and *F*_species_ = 80.2, *p* < 0.001) and at night (*F*_flow treatment × species_ = 26.6, *p* < 0.001; *F*_flow treatment_ = 531.1, *p* < 0.001; and *F*_species_ = 296.8, *p* < 0.001).

### 3D-CLSM images and multifractal analysis

**Figure 3:**
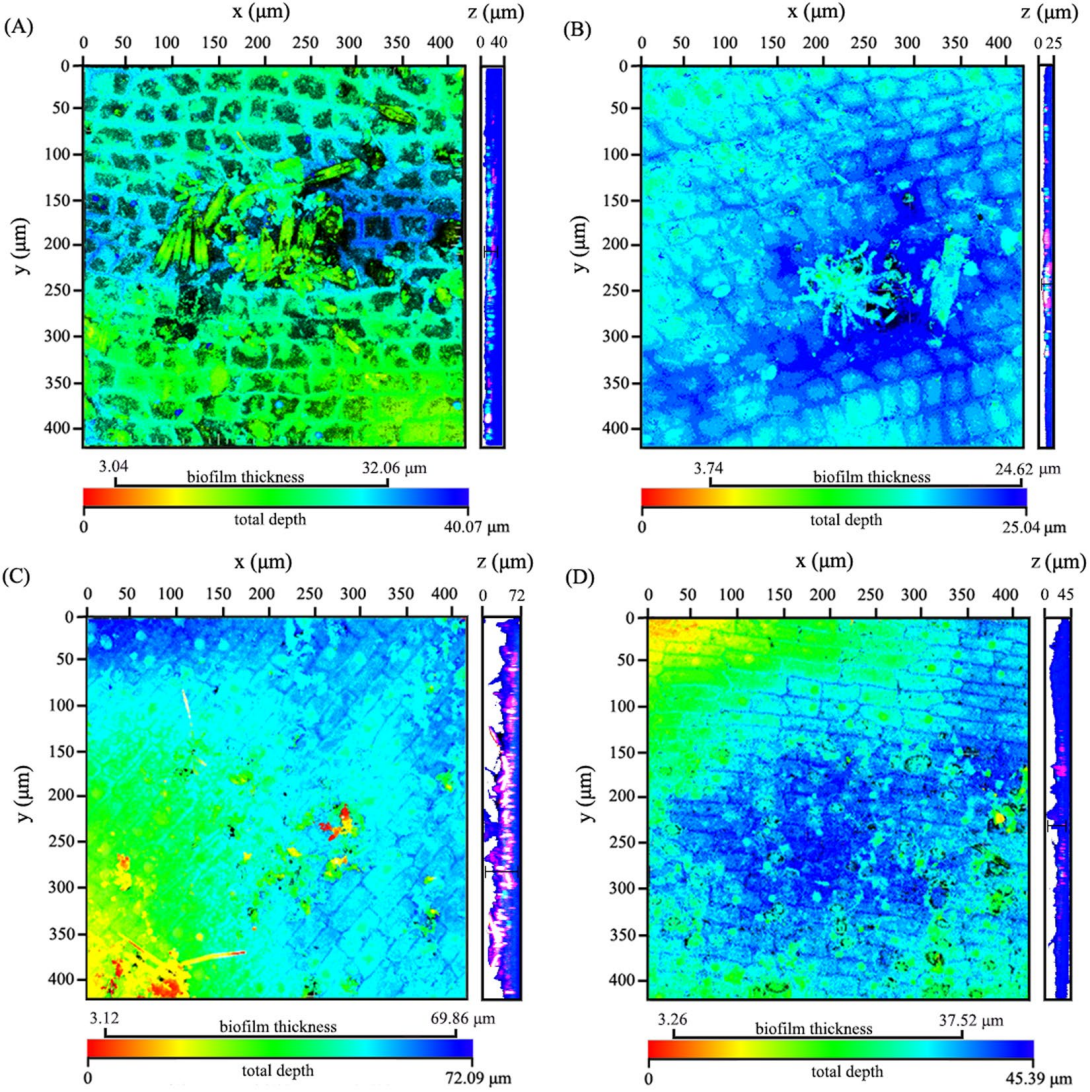
CLSM 3D-depth pictures (left) and the corresponding profile views (right) of the epiphytic microbes on the leaves of *V. natans* (**A** in static water; **B** in flowing water) and *H. verticillata* (**C** in static water; **D** in flowing water). Colors in the 3D-depth pictures indicate the depth, as shown by the bar below the graph. Colors in the profile views indicate the microbes (blue), EPS (red), algae (pink) and chloroplast (cyan) to show legible ranges of the biofilms. Data of biofilm thickness was measured on the profile views and was also put on the bar to better clarify the position of biofilms in depth.

A confocal laser scanning microscope (CLSM) was employed to analyze the profiles of the biofilms attached to the leaves of the submerged macrophytes after they were stained with 4′,6-diamidino-2-phenylindole (DAPI) and the lectin concanavalin A (Con A)-Texas Red, and 3D-depth pictures were obtained (Fig. 3). Epiphytic microbes, including algae and extracellular polymeric substance (EPS)-like aggregates, can be observed at the intercellular furrow on the leaf surface of the two species of submerged macrophytes. The algae/biofilm pixel ratios of CLSM images from *V. natans* were 14.8% ± 0.047 in the static water and 4.2% ± 0.009 in the flowing water; and for *H. verticillata*, 16.8% ± 0.031 in the static water and 9.6% ± 0.012 in the flowing water were obtained. The results showed that algae contributed a small portion of the total area of biofilm. And the relative area of algae in biofilms was affected by flow treatment and host species individually but not their interaction, based on the two-way ANOVA analysis (*F*_flow treatment × species_ = 1.4, *p* = 0.263; *F*_flow treatment_ = 6.3, *p* = 0.027; and *F*_species_ = 37.8, *p* < 0.001). The biofilm thickness ranged from 0 to 29 μm (16.14 ± 7.41 μm, mean ± s.d., *n* = 64) and from 0 to 17 μm (10.73 ± 5.44 μm, mean ± s.d., *n* = 64) on leaves of *V. natans* in the static tank and the flowing tank, respectively, and for *H. verticillata*, it ranged from 0 to 67 μm (34.40 ± 16.93 μm, mean ± s.d., *n* = 64) and from 0 to 34 μm (10.73 ± 5.44 μm, mean ± s.d., *n* = 64), respectively. The two-way ANOVA analysis showed that both water flow and host species influenced the thickness of epiphytic biofilm (*F*_flow treatment × species_ = 19.8, *p* < 0.001; *F*_flow treatment_ = 72.6, *p* < 0.001; and *F*_species_ = 86.5, *p* < 0.001).

**Figure 4:**
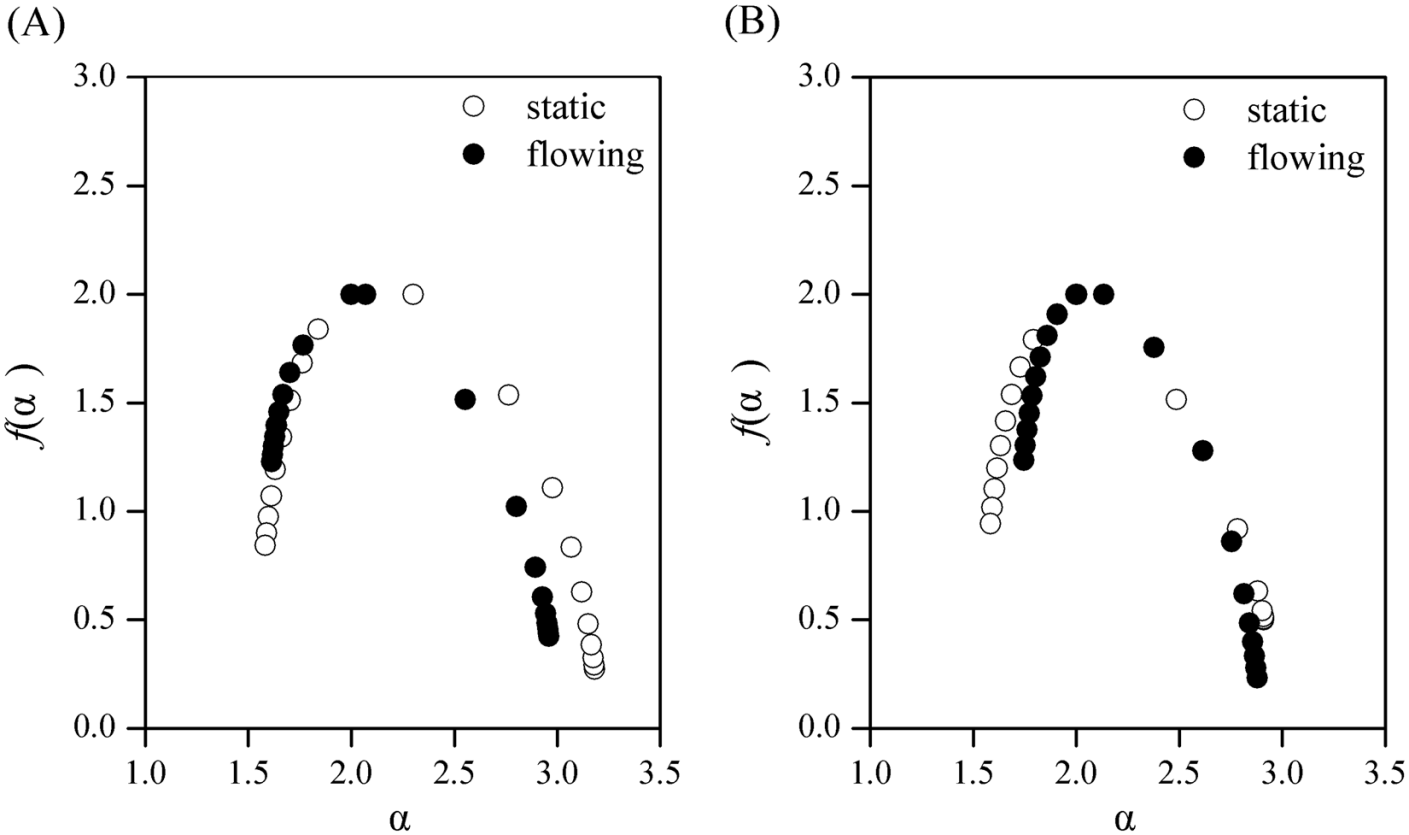
Multifractal spectra of structural characteristics of epiphytic biofilms from different velocities. (**A**) Samples from *V. natans*; (**B**) samples from *H. verticillata*.

To gain a better understanding of biofilm distribution, a multifractal analysis was performed based on the 3D-CLSM images to analyze the topographic characterization of biofilms attached to the leaves of the two species of submerged macrophytes. According to the ln χ_*q*_(ε)-ln ε graphs (Supplementary Fig. 4), partition functions χ_*q*_(ε) and ε had favorable linear correlations (*R*^2^ > 0.98) in dual-logarithm coordinates, which indicated that the surface structures of biofilm had obvious multifractal characteristics. The surface structure characteristics of biofilms were depicted by the α-*f*(α) multifractal spectrum (Fig. 4) and their important parameters are shown in Supplementary Table 1. In the multifractal analysis, a random multifractal must have preferable linearity in the ln χ_*q*_(ε)-ln ε curve plot, and all moments of *q* should have strict linearity that can tend to zero infinitely (ln ε → -∞)^26^. The values of Δα can quantitatively describe the degree of heterogeneity of the biofilm distribution probability in biofilms because the α_max_ and α_min_ are, respectively, the variables of singularity indices of the biggest and smallest biofilm area distribution rules with the changes in ε. The multifractal singular spectrums were all asymmetrical upper convex curves, presenting typical right deviation multifractals, which indicated that most of the patches of biofilm were small. The values of Δα_*s*_ and Δα_*f*_ were 1.60 ± 0.028 and 1.35 ± 0.189 for *V. natans*, respectively, whereas the values of Δα_*s*_ and Δα_*f*_ were 1.33 ± 0.018 and 1.14 ± 0.029 for *H. verticillata*, respectively (Supplementary Table 1). The two-way ANOVA analysis showed that the biofilm heterogeneity was affected by both flow treatment and host species (*F*_flow treatment × species_ = 6.9, *p* = 0.022; *F*_flow treatment_ = 409.3, *p* < 0.001; and *F*_species_ = 336.5, *p* < 0.001).

The $f$(α_min_) and $f$(α_max_) showed the occurrence of microbes that were contained in the minimum and maximum subsets of the biofilm distribution probability, respectively. The difference in the fractal dimensions between the maximum probability (α = α_min_) and the minimum probability (α = α_max_) can be explained by Δ*f* (Δ*f = f*(α_min_) − *f*(α_max_)). Values Δ*f* < 0 and Δ*f* > 0 demonstrate that small probability subsets predominate and large probability subsets predominate, respectively. The two-way ANOVA analysis showed that values of *F*_flow treatment × species_, *F*_flow treatment_ and *F*_species_ were 173.8, 12.5 and 1015.9 (*p* < 0.005), respectively. All Δ*f* (>0) values of biofilms in the flowing water were larger than that in the static water (Supplementary Table 1), suggesting that the distribution probability of a single colony on plant leaves was higher under flow environment than that under static environment.

### 454 pyrosequencing analysis of the bacterial community in biofilms and sediments

After 454 pyrosequencing of 16 S rRNA genes from biofilm and sediment samples, normalized clean reads (7861 reads) were analyzed and operational taxonomic units (OTU) numbers (ranged from 708 to 1257) and Shannon Index values (ranged from 5.2502 to 6.0723) were obtained (Supplementary Table 2). The highest OTU number was detected in unvegetated sediment (1257) in static water.

**Figure 5:**
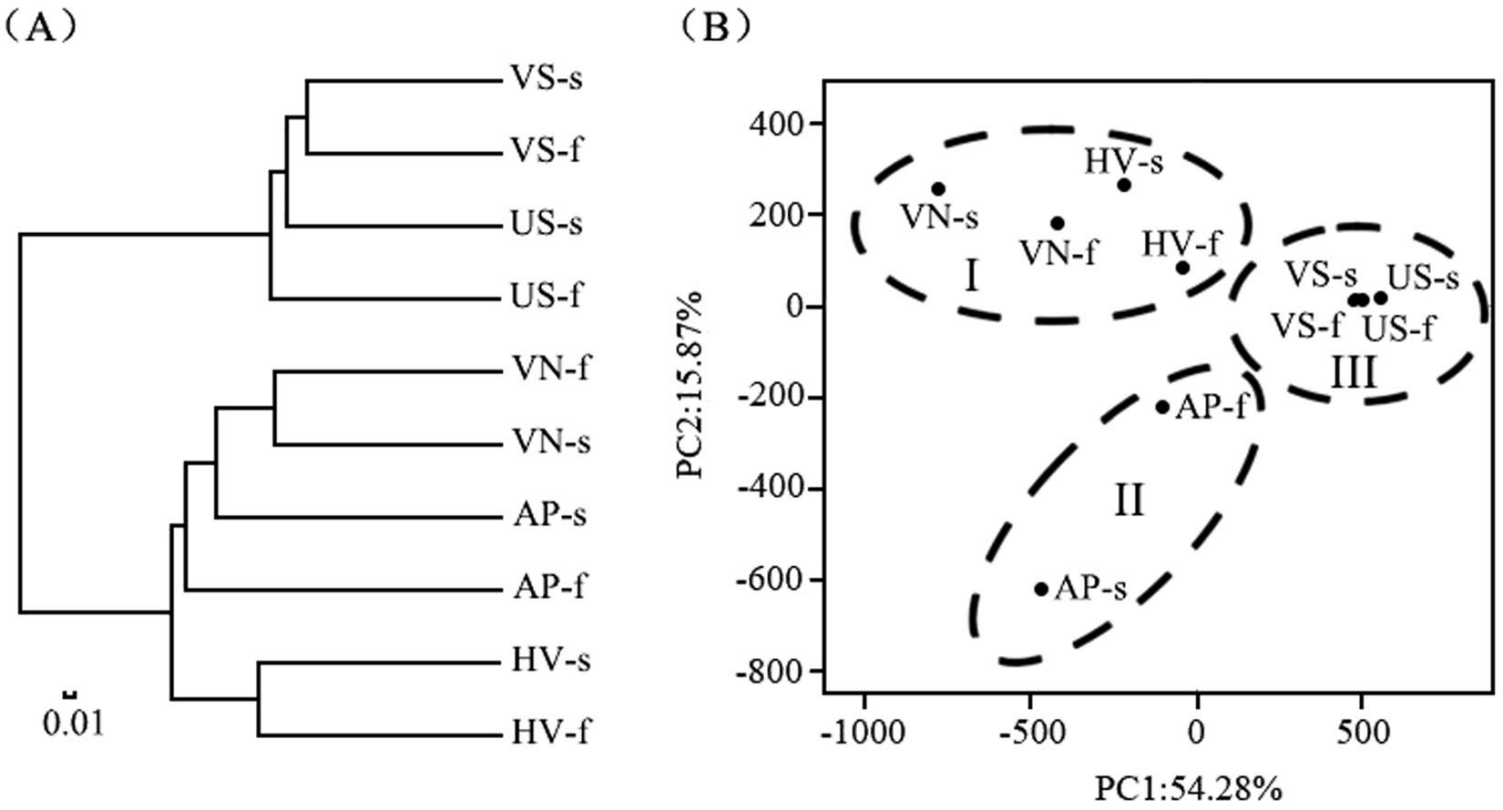
Cluster analysis (based on Bray–Curtis distances) (**A**) and principal coordinate analysis (**B**) at the OTU level. VN: *V. natans*; HV: *H. verticillata*; AP: artificial plant; -s: in static water; -f: in flowing water; VS: vegetated sediment; US: unvegetated sediment.

As revealed by the cluster analysis based on Bray-Curtis distances at the OTU level (Fig. 5A), three main groups were generated from 10 samples. Sediment samples were clustered in group I, and biofilm samples from *V. natans* and artificial plants were in group II, whereas biofilm samples from *H. verticillata* were clustered in group III. Two main components from the principal coordinate analysis (PCoA) explained 70% of the total variance (Fig. 5B). Three groups were generated, including group I (four biofilm samples from *V. natans* and *H. verticillata*), group II (two biofilms from artificial plants) and group III (four sediment samples).

### Characterization of the microbial community in biofilms and sediments

**Figure 6:**
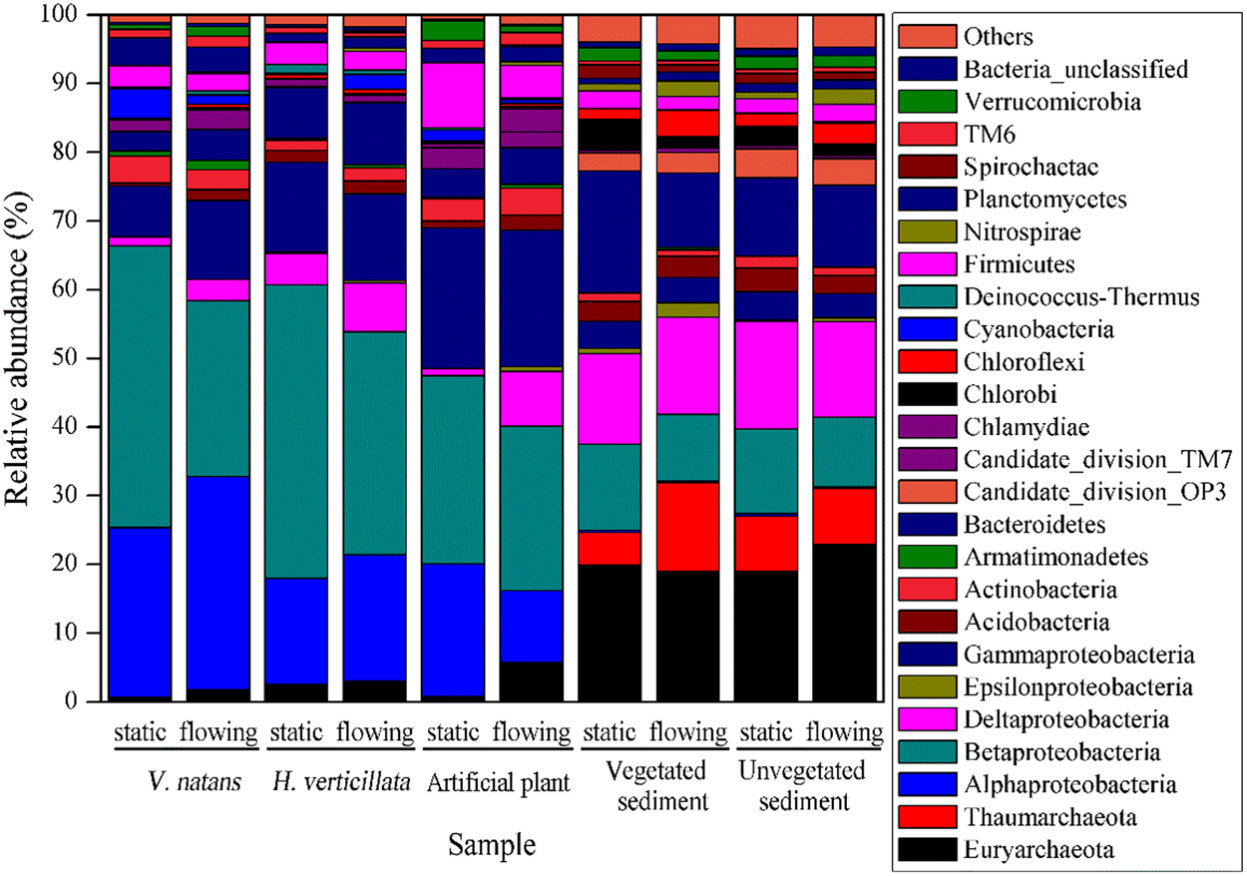
Relative abundances of phyla or classes in microbial communities from all the samples. Names of main phyla or classes (relative abundance >1% in any sample) are shown in the figure.

Except unclassified archaea and bacteria, a total 46 phyla were detected in at least one sample, and 16 phyla, shared by all the samples, accounted for 91.6% of all the classified sequences. In biofilm samples, 2 archaea phyla and 35 bacteria phyla were detected at least once, with 3 archaea phyla and 42 bacteria phyla detected in at least one sediment sample. The relative abundance of archaea in sediments was much greater than that in biofilms. *Proteobacteria* (63.66–77.11%) was the most dominant phylum in biofilm, followed by *Firmicutes* (2.54–9.55%), *Bacteroidetes* (2.75–9.03%), *Planctomycetes* (1.20–4.07%) and *Actinobacteria* (1.44–4.01%) whereas the primary phyla in sediments were *Proteobacteria* (28.50–33.10%), *Euryarchaeota* (18.93–22.83%), *Bacteroidetes* (10.88–17.73%) and *Thaumarchaeota* (4.80–12.89%) (Supplementary Table 3). All the phyla in biofilms could be found in the sediments (Fig. 6).

The dominant classes in *Proteobacteria* were *Alpha-* and *Beta-proteobacteria* in biofilms and *Delta-proteobacteria* in sediments. In general, water flow increased the relative abundance of *Methanobacteria* and *Methanomicrobia* in phylum *Euryarchaeota*, *Acidobacteria* in phylum *Acidobacteria*, *Cytophaga* in *Bacteroidetes*, *Chlorobia* in *Chlorobi*, *OM190* in *Planctomycetes*, *Delta-proteobacteria* in *Proteobacteria*, *Nitrospira* in *Nitrospirae*, *Gemmatimonadetes* and *Armatimonadetes* but decreased that of *Planctomycetacia* in *Planctomycetes* and *Beta-proteobacteria* in *Proteobacteria* in biofilms (Supplementary Table 3).

**Figure 7:**
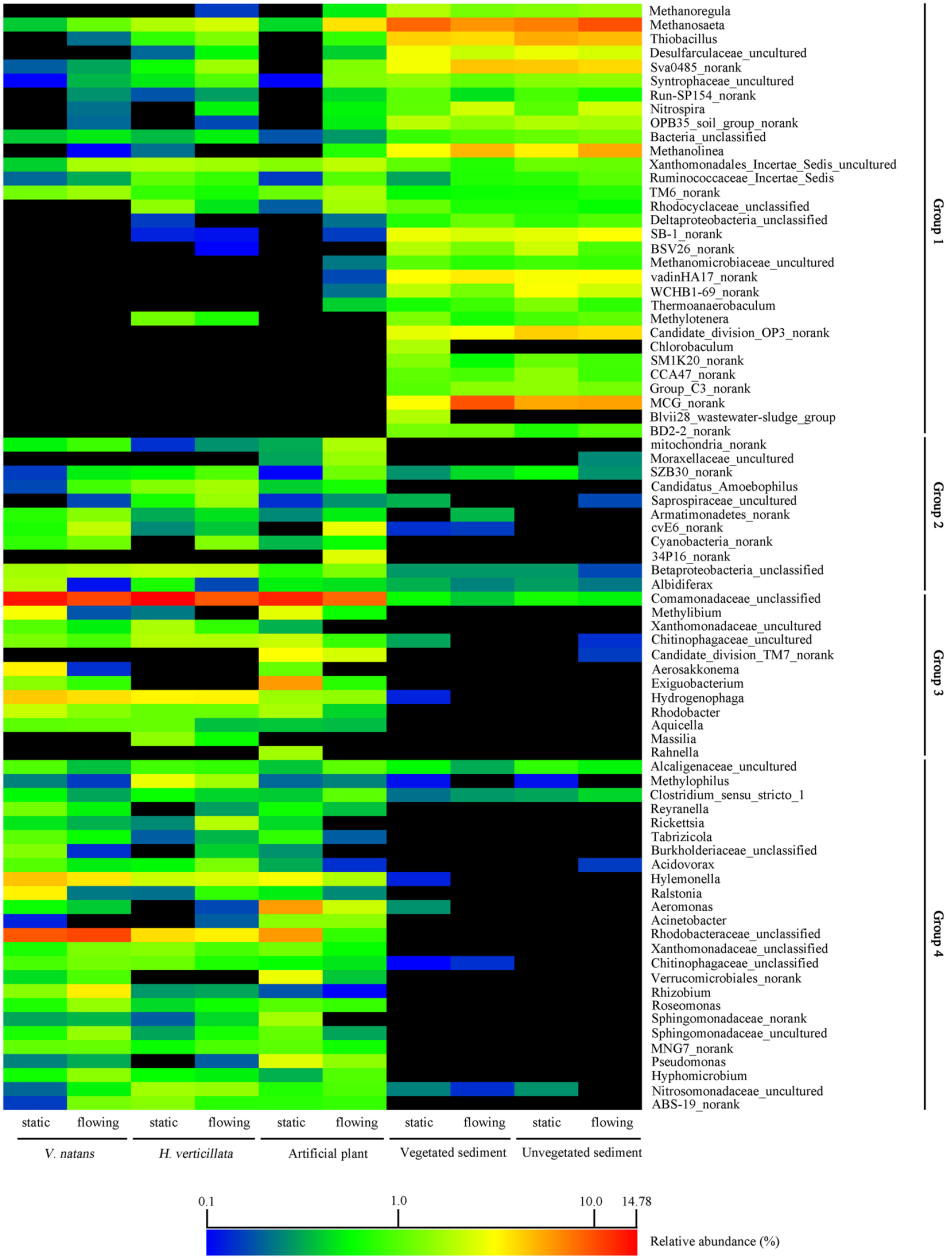
Relative abundances (%) of the dominant genera (>1% in at least one sample) in biofilm and sediment samples. Percentages of genera below 0.1% are marked with black.

A total of 750 genera were identified in this study and 65 genera (43.47% of the classified sequences) were shared by all 10 samples. A heat map was generated to analyze the distribution of 78 genera occurring at > 1% in at least one sample (Fig. 7). According to the distribution of the relative abundances, the selected genera were divided into the following 4 groups: Group 1, dominant genera in sediments; Group 2, genera of higher abundance in biofilms under flowing water than in the static environment; Group 3, genera of lower abundance in biofilms under flowing water than in the static environment; and Group 4, other dominant genera in biofilms.

## Discussion

In natural environments, aquatic plants are always affected by water flow through direct effects (stretching, breakage, uprooting, etc.) and indirect effects (changes in gas exchange, bed material distribution, sediment resuspension, etc.)^18^. In this study, water flow inhibited the growth of *V. natans* and *H. verticillata* (Fig. 1A) and plants also altered the vertical distribution of water velocity. There might be differences in plant distribution under varied flow regimes. For example, a previous investigation showed that the occurrence and abundance of aquatic macrophytes decreased with increased current velocity, and survey units with no visible flow or fast flow were characterized by different indicator species^27^. A recent report^28^ showed that flow turbulence (1.06–1.93 cm s^−1^) could induce oxidant stress, inhibit plant growth and photosynthetic efficiency and decrease the carbon content in the tissue of the submerged macrophyte *Chara fibrosa*. Differences in macrophyte morphology have been related to N uptake, and a higher leaf perimeter-to-area ratio has been associated with higher uptake rates^29^. However, *V. natans* have a lower leaf perimeter-to-area ratio than *H. verticillata*, because compared to *V. natans*, *H. verticillata* has more leaves and lower single leaf area. The difference in plant growth may be ascribed to their adaptions to nutrient availability because *H. verticillata* always grows rapidly in high nutrient environment.

Apart from the dissolved oxygen content, the distribution of oxygen can also affect microbial activities and community structures and further influence the efficiency of pollutant removal to a large extent^30^. Oxygen can be released from photosynthetic systems of submerged plants under light. The oxygen concentrations in leaf tissues of two species of plants were lower in the water flow than in the static water during the daytime (Fig. 2). The reduced oxygen concentration in plant leaves of submerged macrophytes in the flow may be ascribed to the decreased net photosynthesis and increased respiration^31^. As water flow decreased biofilm densities in this study (Fig. 3), the reduction in light intensity to the leaves caused by biofilms was not the key factor inhibiting the photosynthetic reaction and plant growth of the two species of submerged macrophytes. The sharper gradient of phyllosphere oxygen concentrations in the flowing water illustrated that water flow promoted the exchange of oxygen between leaf and water. Previous study^32^ illustrated that biofilms contributed a lot to phyllosphere oxygen profiles of stems and senescent leaves of *Potamogeton malaianus*, but the rate of contribution was minor to young leaves due to the high oxygen release of young leaves and thin biofilms. In the present study, biofilms on young leaves were sampled to explore the effect of water flow to the biofilms. Algae occupied a small portion of the total area of biofilms, and the maximum biofilm thickness detected in this study was approximately 67 μm, which might have minor contribution to the total detection depth (3000 μm). Thus the biofilm on young leaves might have a small contribution to the oxygen profiles as reported by Dong *et al*.^32^.

The leaves of submerged macrophytes are ideal substrates for biofilm growth^5^. Unlike abio-substrates (including riverbed and sediment), submerged macrophytes are flexible and can oscillate in the stream water. Biofilms in streams are also different from those grown in the laboratory because they are continuously exposed to a diverse inoculum that includes bacteria, archaea, algae, fungi, protozoa and even metazoan and are sometimes subjected to disturbance by water flow^33^. To date, the effects of water flow on biofilms has been focused on the abio-substrates; however, little is known about the effects of water flow on biofilms attached to submerged macrophytes. Excessive shear force caused by water flow would destroy the structure of formed biofilms and result in biofilm deformation, breakup and detachment^34^. As revealed by CLSM images, water flow obviously reduced the thickness of biofilms (Fig. 3). Recent investigations have found the formation of thinner yet denser biofilms under high and turbulent flow regimes in the drinking water distribution system, in comparison to the more porous and loosely attached biofilms at low flow rates^23^. Battin and his collaborators found that slow-flow contributed to an increase of biofilm thickness, surface sinuosity and density^15^. A recent report showed that shear force from water flow led to an equilibrium between biofilm thickness and density, resulting in a steady-state structure^35^.

Results from multifractal analysis suggested that the heterogeneity of biofilms in the flowing water was lower and has higher distribution probability of a single colony than that of biofilms in the static water, demonstrating that water flow altered the biofilm surface area. Current velocity affected the architecture and dynamics of natural, multiphyla, and cross-trophic level biofilms from a forested piedmont stream^15^. Recently, a 3-D biofilm model was used to illustrate the effects of water flow on biofilms in a water channel and found that excessive shear force could detach or bend the thin-necked biofilm colony (a common structure during biofilm formation), decreasing the biofilm thickness and roughness^36^. The heterogeneity of biofilms was also affected by surface characteristics of plant leaves. Leaf surface of *V. natans* was rougher than that of *H. verticillata* (Fig. 3), which might be the reason why biofilms on leaves of *V. natans* showed stronger heterogeneity in the present study.

According to the rarefaction curves (Supplementary Fig. 6), the reads number 7861 suggested a sufficient sequencing depth in the present study. Bacteria compositions in biofilms and sediments samples at the OTU level were determined by the type of substrate (Fig. 5A and B), which was in agreement with previous reports that the bacterial communities showed host-specificity^37,38^. The dominant phyla/classes in this study (Fig. 6) were also detected in stream biofilms, sediments, river and biofilms attached to submerged macrophytes^33,37,38^. As revealed by the cluster analysis (Fig. 5A), the dominant phyla/classes in biofilms were different from those in sediments, in agreement with our previous report^25^. The Fig. 5A illustrated further information that bacterial communities in biofilm on artificial plants were largely affected by the inoculating source (the *V. natans* in the same system with artificial plants) rather than the water flow. The percentages of cyanobacteria in this study were significantly lower than those in biofilms from submerged plants in eutrophic Lake Taihu, China^9^. The low cyanobacteria concentrations in this study might be ascribed to low concentration of total nitrogen and phosphorus and the difference in plant types and bacterial primers. *Euryarchaeota*, *Delta-proteobacteria*, *Bacteroidetes*, *Beta-proteobacteria* and *Thaumarchaeota* in sediments in this study were also dominant in freshwater sediment from Lake Dianchi, China^39^ and Kinneret, Israel^40^.

A total of 248 genera (12.82% of the classified sequences) and 150 genera (4.88% of the classified sequences) were only found in biofilm samples and sediments, respectively, suggesting that most of genera detected in this study can inhabit both biofilms and surficial sediment. The differences in bacterial composition between biofilms and sediment sources might be ascribed to the environmental parameters and nutrient availability^41^. For example, *Thaumarchaeota* and *Chlorobi* were barely found in biofilm samples because the demand for anaerobic environment and sulfur^42,43^; and low abundance of *Cyanobacteria* in sediments may be ascribed to the low light^44^ (Fig. 6).

Over the past two decades, research has shown the importance of biodiversity in biological systems, and microbial communities with higher richness (the number of species in a community) were found to have higher functionality and stability than microbial communities with lower richness^45^. It is interesting to note that water flow promoted epiphytic microbial diversity in biofilms in terms of OTU numbers and Shannon Index values (Supplementary Table 2). Metacommunity theory proposes that the composition and diversity of ecological communities are shaped by the interplay between regional dispersal dynamics, the local environment and biotic interactions^33^. Sediments can be a source of bacteria in biofilms, as water flow can cause the suspension of sediments and carry particles^18^. For example, water flow increased the abundances of the classes *Methanomicrobia* and *Delta-proteobacteria* (Supplementary Table 3), and genera (Fig. 7) inhabiting sediments increased in biofilms attached to the three types of substrates. A recent report^4^ showed that moderate water velocities (5–45 cm s^−1^) significantly enhanced the trapping and retention of fine particles by submerged macrophytes *Potamogeton pectinatus*, *M. spicatum* and *Ceratophyllum demersum*. Fine particles provide very diverse habitats on a small scale, which favor biodiversity^46^. Our results also support the viewpoint that hydraulics drives the distribution of microbial diversity^41^.

Another possible reason that explains the biodiversity is the allelopathic exudates and architectural complexity of plants^4^, especially at the initial stages of bacteria attachment and subsequent settlement^47^. There were complex topographical characterizations on submerged macrophytes used in this study and on the same type of artificial plants in our previous report^5^. The host-specified epiphytic bacterial communities in the present study suggested a great influence of plant activity. The lower microbial density on leaves of *H. verticillata* might be induced by the inhibiting effect of the allelopathic exudates. A higher architectural complexity would promote the trapping and retention of fine particles^4^, which might have more contribution to the epiphytic bacterial diversity of *H. verticillata* and the artificial plant than *V. natans* in the flowing water.

As water flow can shape the oxygen profiles around the leaf-biofilm interface, it might also dilute the allelopathic exudates produced by plants and/or microbes in biofilms. In addition, adhesive properties, extracellular polymeric substances and extracellular bacterial structures (such as flagella, pili and fimbriae) of microbes may also affect the bacterial composition of biofilms under water flow^22^. A recent report^48^ demonstrated that algal periphyton diversity attached to submerged macrophyte can be affected by its hosts. As revealed by CLSM images, the thickness and extracellular polysaccharides content (marked by lectin ConA-Texas-Red conjugate) of biofilms decreased (Fig. 3). Our results demonstrated that flow and intrinsic attributes of plant shaped the development of biofilm architecture and community composition in this study, while a shift from water flow control to coupled biophysical controls occurred on the bacterial community in stream biofilms^49^.

In this study, we explored the influence of water flow on submerged macrophyte-biofilm systems and found that the water flow inhibited the growth of submerged macrophytes and changed the oxygen profiles at leaf-biofilm interface. Water flow decreased the densities and roughness of biofilms. The probability of a single colony-like biofilm was higher on leaves in water flow than in static water. The bacterial compositions in biofilms were different from those in sediments, and substrate types mainly determined the bacterial community. However, water flow can increase bacterial biodiversity in biofilms. These results suggested that water flow can be used to regulate the biomass, distribution and bacterial diversity of epiphytic biofilms, which in excess may cause negative impacts on submerged macrophytes in CWs.

## Methods

### Experiment facility and treatment conditions

To investigate the effects of water flow on submerged macrophyte-biofilm systems under similar water quality, a tank (length × width × depth was 2000 × 1000 × 500 mm)-pump-open channel (length × width × depth was 2000 × 300 × 500 mm) cycling system (Supplementary Fig. 1) under a glass shelter was employed in this study. There was a treatment tank and a treatment channel with two regulating tanks in the cycling system. Approximately 1200 L of water and 8 cm of sediments (collected from Lake Wulongtan, Nanjing, China) were used in this system.

The water depth was approximately 35 cm in both the tank and the channel. Current velocities were measured by an acoustic Doppler velocimeter (Flow tracker ADV, YSI, USA). Velocities in the sampling zones of the static tank and flow channel were 0–0.1 cm s^−1^ and 4.5–5.5 cm s^−1^, respectively. Healthy plants (from Gaochun aquatic plant cultivation base, Nanjing, China) with similar length and biomass were selected, cleaned and then incubated in the plant zones. The incubation time was 21 days. (See more details in the Supplementary Information.)

During the flow treatment, concentrations of TN and TP in the water were determined using an AA3 flow continuous chemistry analyzer (SEAL, Germany) between 8.30 and 10 a.m. each day. Then TN and TP concentrations were adjusted to approximately 2 mg L^−1^ with KNO_3_ and approximately 0.05 mg L^−1^ with NaH_2_PO_4_, respectively. Values of pH, dissolved oxygen, electronic conductivity and oxidation-reduction potential in the center of both sampling zones were determined using a HQ30d portable multi-parameter digital analyzer (HACH, USA) between 9 and 9:30 a.m. each day. Current velocities in the plant zones were measured at the same time.

### Oxygen determination and sample collection

Puncture test with oxygen macro-sensors was an effective tool usually used to characterize oxygen fluxes in higher-plant cells under abnormal or stressed states^50^. The puncture tests were used to detect the *in situ* oxygen profiles at the leaf-water interface using a micromanipulator MM33–2 meter system (Unisense, Denmark) with a needle oxygen sensor (tip size 10 μm). The average slope of the oxygen gradient was calculated with the change rate of oxygen with depth. Six replicates were conducted for each treatment of each plant species. (See more details in the Supplementary Information.)

At the end of experiment, to avoid the impacts of sediments, healthy leaves at 10–20 cm below the water surface were collected for biofilm collection. Leaves at 10–15 cm below the water surface were used for the CLSM analysis. The surficial sediments (0–5 cm depth) were collected from the *V. natans* vegetated or unvegetated zones of both the static tank and flow channel and kept at −80 °C. Three replicates were conducted for each treatment of each plant.

### Biofilm detach and microbe cell counting

The epiphytic microbes on leaves were detached with 10 mL of a cold phosphate buffer solution (PBS, 0.1 M, pH 7.2) with 3 g of glass beads using 3 min ultra-sonication, 30 min of shaking (225 r min^−1^) and subsequent ultra-sonication for 3 min^2^. The eluents were immediately fixed in a final concentration of 2% formaldehyde. The area of the leaves was measured as reported in a previous study^51^.

One hundred microliters of eluent was mixed with 700 μL DAPI solution (10 μg mL^−1^; Roche, Germany), kept in the dark for 30 min, and filtered through a 0.22 μm filter. The number of blue spots on the membrane was examined under a fluorescence microscope (Zeiss, Germany). Samples for DNA extraction were detached from 20 leaf samples in the same way, the eluents were centrifuged at 8000 r min^−1^ for 10 min and the solids were collected and saved in ethanol at −80 °C. Samples of artificial plants were used as a control. Three replicates were conducted for each treatment of each plant.

### Microscopy analysis

For the CLSM analysis, fluorescent dye DAPI and lectin Con A-Texas-Red conjugate (Invitrogen, USA) were employed to mark the DNA and the extracellular polysaccharides, respectively. Briefly, leaves were cut into small pieces (5 mm × 5 mm) and stained by a 10 μg mL^−1^ DAPI solution for 45 min. After being washed with a 0.1 M PBS solution (pH 7.2) 3–5 times, samples were further stained with 10 μg mL^−1^ of a ConA-Texas Red solution for 30 min. Then, the stained leaves were visualized under a CLSM (Zeiss LSM 800, Germany) after the second washing process. The generated pictures were processed and 3D-depth pictures and profile views were composed using the software ZEN 2 2013 (Zeiss, Germany). Four replicates were conducted for each treatment of each plant. (See more details in the Supplementary Information.)

The topmost and deepest layers with fluorescence colors of biofilm were located in the profile view, and the interval between was calculated as the total biofilm thickness of the single image. To calculate the average biofilm thickness, the 3D-depth picture was equally divided into 5 × 5 grids with Photoshop CS6 (version 13.0.0.0). The cross points of the boundary lines were set to be measuring points. The interval between the topmost and deepest layers with fluorescence colors of biofilm at measuring point was calculated as biofilm thickness. Total 16 measuring points were set on each 3D-depth picture and 4 replicates of each treatment were conducted. The relative area of algae in biofilm was calculated with 3D-depth pictures using Photoshop CS6 (version 13.0.0.0) according to Chen *et al*.^51^. The range of algae was covered by black color and the left of the biofilm was covered by white color. Then the pixel counting was conducted and the relative area of algae in biofilm was calculated by the ratio of black pixels to the total pixels of the whole picture. 4 replicates of each treatment were conducted.

### Multifractal analysis

In general, multifractal describes a measurement that is defined in a certain area or volume and can decompose the defined domain into a series of subdomains in space, and according to the singularity of this measurement, every subdomain constitutes a single fractal. Box counting is one of the methods in a multifractal analysis that is used to count the number of occupied boxes over a range of different box sizes of images. In this study, the box counting method was applied according to our previous report^52^. The width of the multifractal spectrum is Δα. The difference in the fractal dimensions between the maximum probability (α = α_min_) and the minimum probability (α = α_max_) is expressed as Δ*f* (Δ*f* = *f*(α_min_) − *f*(α_max_)). Four replicates were used for each treatment of each plant in this study. (See more details in the Supplementary Information.)

### 454 pyrosequencing and data processing

Microbial DNA was extracted from biofilms and sediment samples of three replicates using a FastDNA SPIN Kit for Biofilm/Soil (MPBIO, USA) according to manufacturer’s protocols. Aliquots of DNA samples from triplicates of the same treatment were combined for PCR analysis. The primers 799 f and 1492r^53^ of bacterial 16 S ribosomal RNA gene were used to amplified the DNA fragments in a 20 μL mixture containing 4 μL of 5 × FastPfu Buffer, 2 μL of 2.5 mM dNTPs, 0.8 μL of each primer (5 μM), 0.4 μL of FastPfu Polymerase and 10 ng of template DNA. The PCR protocols were 95 °C for 2 min, followed by 25 cycles at 95 °C for 30 s, 55 °C for 30 s, and 72 °C for 30 s and a final extension at 72 °C for 5 min.

PCR products purified by AxyPrep DNA Gel Extraction Kit (Axygen Biosciences, USA) were quantified using QuantiFluor™ -ST (Promega, USA). A mixture of amplicons was used for pyrosequencing on a Roche 454 GS FLX + Titanium platform (Roche 454 Life Sciences, USA) according to standard protocols.

In total, 136987 raw reads were obtained from all 10 samples (Supplementary Table 2). The resulting sequences were processed using QIIME (version 1.17). After removing sequences with average quality scores < 20 over a 50 bp sliding window and sequences shorter than 200 bp with homopolymers longer than six nucleotides and containing ambiguous base calls or incorrect primer sequences, a total of 109430 high-quality sequences were produced with an average length of 411 bp. To analyze these cross-talking sequences, the effective sequences of each sample were submitted to the RDP Classifier^54^ to identify the archaeal and bacterial sequences.

To fairly compare the 10 samples at the same sequencing depth, the sequence number was normalized to the minimal sequence number 7861 among these samples (Supplementary Fig. 6). OTUs were clustered with a 97% similarity cutoff using UPARSE (version 7.1 http://drive5.com/uparse/), and chimeric sequences were identified and removed using UCHIME. The phylogenetic affiliation of each 16 S rRNA gene sequence was analyzed by RDP Classifier (http://rdp.cme.msu.edu/) against the SILVA (SSU115) 16 S rRNA database using a confidence threshold of 70%^55^. The heat map of relative abundance of the dominant genera was generated using the software HemI^56^ (version 1.0 http://hemi.biocuckoo.org/).

### Statistical analysis

Statistical differences were determined by two-way ANOVA using the software SPSS (version 19.0). A Bray-Curtis index of dissimilarity cluster analysis based on OTUs was conducted using the statistical software PAST (version 3.10). Principal coordinate analysis was carried out using CANOCO (Version 4.5) based on OTUs.

### Data availability

The authors declare that all data supporting the findings of this study are available within the article and its Supplementary Information files or from the corresponding author by reasonable request. All sequence data are available through the NCBI, with project accession number PRJNA299092.

## Electronic supplementary material

Supplementary information
