# Supplementary material for: Effects of water flow on submerged macrophyte-biofilm systems in constructed wetlands
Source: Sci Rep. 2018 Feb 8;8:2650. doi: 10.1038/s41598-018-21080-y (PMC5805772; doi:10.1038/s41598-018-21080-y)
Supplement: Supplementary file 1 — Supplementary information [file 41598_2018_21080_MOESM1_ESM.pdf]

# Effects of water flow on submerged macrophyte-biofilm systems in constructed wetlands

Bing Han[1], Songhe Zhang[1,*], Peifang Wang[1,*], Chao Wang[1]

[1]Ministry of Education Key Laboratory of Integrated Regulation and Resource Development on Shallow Lakes, College of Environment, Hohai University, Nanjing 210098, China.

[*]Corresponding authors

Email addresses: shzhang@hhu.edu.cn; pfwang2005@hhu.edu.cn

# Supplementary Methods

**Experiment facility and treatment conditions.** As the Supplementary Fig. 1 B shown, the depth of the overlying water was 35 cm in the flume F, and 8 measuring points of the current velocities were evenly set at the vertical axe of each cross section (A to G). The results (Supplementary Fig. 3A) showed that, from the depth of 10 cm to 20 cm of cross section E, F and G, the velocities were much similar (4.5-5.5 cm $s^{-1}$). The average value of the velocities ($n$ = 9) was 5.04 cm $s^{-1}$. Whereas in the flume S, the average velocity is 0.01 cm $s^{-1}$, with a significant difference ($p < 0.001$). So the flow regime of flume F was considered to be flowing, with that of flume S to be static. The flume S and F were labeled as static tank and flow channel, respectively. The plant zone was set between cross section E and G, and the sampling zone was set at water depth 10-20 cm.

Besides, the *Re* values of the middle cross sections (cross section $F_s$ and $F_f$) in the plant zones were calculated by the formula:

$$Re = \frac{2\rho v R}{\mu} \qquad (1)$$

where $\rho$ is the water density, $v$ is the average velocity of the cross section, $R$ is the hydraulic radius of the cross section and $\mu$ is the dynamic viscosity of water. During the experiment, the water temperature ranged from 28.2-31.2 °C, so the parameters of water at 30 °C were selected as $\rho$ = 995.7 kg $m^{-3}$ and $\mu$ = 0.797×$10^{-3}$ Pa*s. And $v_{Fs}$ = 0.0001 m $s^{-1}$, $R_{Fs}$ = 0.27 m; $v_{Ff}$ = 0.04 m $s^{-1}$, $R_{Ff}$ = 0.16 m, therefore, the *Re* values of the cross section $F_s$ and $F_f$ were 67. 5 and 15991.2, respectively.

Plants were planted in the water flow to avoid the impact of secondary flow generated

by the experimental system. The plant density was 400 plants per square meter and the plant numbers were 144 in the static tank and 48 in the flow channel, respectively. The *V. natans* and *H. verticillata* were incubated in sequence. The artificial plants were set among the *V. natans* plants as a control.

In the flowing channel, the velocity range at water depth 10-20cm in the plant zone was reduced from 4.5-5.5 cm $s^{-1}$ to 2.6-4.7 cm $s^{-1}$ (2.9-4.8 cm $s^{-1}$) by *V. natans* and artificial plants (*H. verticillata*), whereas the velocity range at water depth 0-5 cm was promoted from 5.6-6.3 cm $s^{-1}$ to 5.9-7.4 cm $s^{-1}$ (6.0-7.4 cm $s^{-1}$) (Supplymentary Fig. 2B and C).

Values of pH, dissolved oxygen, electrical conductivity and oxidation-reduction potential determined in the center of both sampling zones illustrated that no significant difference ($p > 0.05$) was found between the water parameters in the static water and those in the flowing water (Supplymentary Table 4).

**Oxygen determination**. Before the tests, the oxygen microelectrode was linearly calibrated from signal readings in oxygen-free and air-saturated fresh water at the experimental temperature. Data under light was gathered between 2 and 4 p.m. whereas data under dark was obtained between 8 and 10 p.m. The motor and object stage of the microelectrode system were set on the vessel wall of the tank/flume (Supplementary Fig. 5). To maintain a still water condition, the pump and valve between the static tank and the flowing channel were shut down during the test on the leaves in the static tank. To avoid the noticeable vibration of the microelectrode caused by water fluctuation during the test on the leaves in the flowing channel, the

surface flow velocity in the flowing channel was adjusted to approximately 1 cm $s^{-1}$. Young leaves of *V. natans* and *H. verticillata* at 10-15 cm below the water surface were used for the puncture test. The leaf was fixed onto the object stage with two small clips (Supplementary Fig. 5) at the water depth of approximately 10 mm. The oxygen microelectrode was fastened on the motorized micromanipulator, and its tip was set manually approximately 1500 μm above the leaf surface. The microelectrode moved vertically via the motorized micromanipulator. The motorized micromanipulator was set to move 300 steps down (10 μm each step) to cover 3000 μm.

**Biofilm detach and microbe cell counting.** The target leaf was put on a piece of white paper on which four endpoints of an equal-length and orthogonal cross are printed. A X-ray illuminator was set under the paper to increase brightness and contrast, and a piece of sheet glass was paved on the leaf to keep it flat. The digital image was captured and then the pixels were counted by the Adobe Photoshop CS6 (version 13.0.0.0). The leaf area was calculated by the ratio of the number of leaf pixels to the number of pixels of the reference facet formed by the four control endpoints.

**Microscopy analysis.** The fluorescence of three channels (DAPI, Texas Red and chloroplast) were captured layer by layer from the highest peak of the biofilm to the interior of epidermis cells till the fluorescence disappeared. The maximum excitation and emission wavelengths of DAPI-stained double stranded DNA are 358 nm and 461

nm, respectively. The maximum excitation and emission wavelengths of Texas Red are 589 nm and 615 nm, respectively. The chloroplast spontaneous fluorescence excited at 488 nm was captured at 650-750 nm. The layer thickness was set at 1.67 μm. The scanning was conducted under 200× and the imaged area was approximately 420 μm × 420 μm.

**Multifractal analysis.** Briefly, the 3D-CLSM images were adjusted to 256 × 256 pixels and divided into many boxes of size ε×ε, where ε = 1/L (L = 256, 128, 64, 32, 4, 2 or 1), using the software Adobe Photoshop CS6 (version 13.0.0.0). To calculate the multifractal spectrum, the value distribution probability of the biofilm in the box $(i, j)$ was expressed as

$$P_{ij}(\varepsilon) = n_{ij}/\sum n_{ij} \qquad (2)$$

where $n_{ij}$ is the biofilm value of the box $(i, j)$ of size ε.

$P_{ij}(\varepsilon)$ can be described as multifractal as

$$P_{ij}(\varepsilon) \propto \varepsilon^{\alpha} \qquad (3)$$

$$N_{\alpha}(\varepsilon) \propto \varepsilon^{-f(\alpha)} \qquad (4)$$

where the exponent α depending upon the box $(i, j)$ is the singularity of the subset of probabilities, $N_{\alpha}(\varepsilon)$ is the number of boxes of size ε with the same biofilm value distribution probability, and $f(\alpha)$ is the fraction dimension of the α subset. The dependence of $f(\alpha)$ on α is the multifractal spectrum. Generally, the fractal

dimension $f(\alpha)$ can be obtained from the partition function $\chi_q(\varepsilon)$ expressed as a power law of $\varepsilon$ with an exponent $\tau(q)$ applied in statistical physics as follows:

$$\chi_q(\varepsilon) = \sum P_{ij}^{q}(q) = \varepsilon^{\tau(q)} \quad (5)$$

where $q$ is the moment order ($-\infty < q < \infty$). $\tau(q)$ can be obtained from the slope of the ln $\chi_q(\varepsilon)$-ln $\varepsilon$ curve, and the generalized fractal dimension $Dq$ is defined as

$$Dq = \tau(q)/(q-1) \quad (6)$$

$f(\alpha)$ can be obtained by performing the Legendre transformation as follows:

$$Dq = d\tau(q)/dq \quad (7)$$

and

$$f(\alpha) = q\alpha(q) - \tau(q) = q\frac{\tau(q)}{dq} - \tau(q) \quad (8)$$

We took the maximum $|q|$ as 10 since $|d\alpha_{max}|/\Delta\alpha$ and $|d\alpha_{min}|/\Delta\alpha$ were all less than 0.1%.

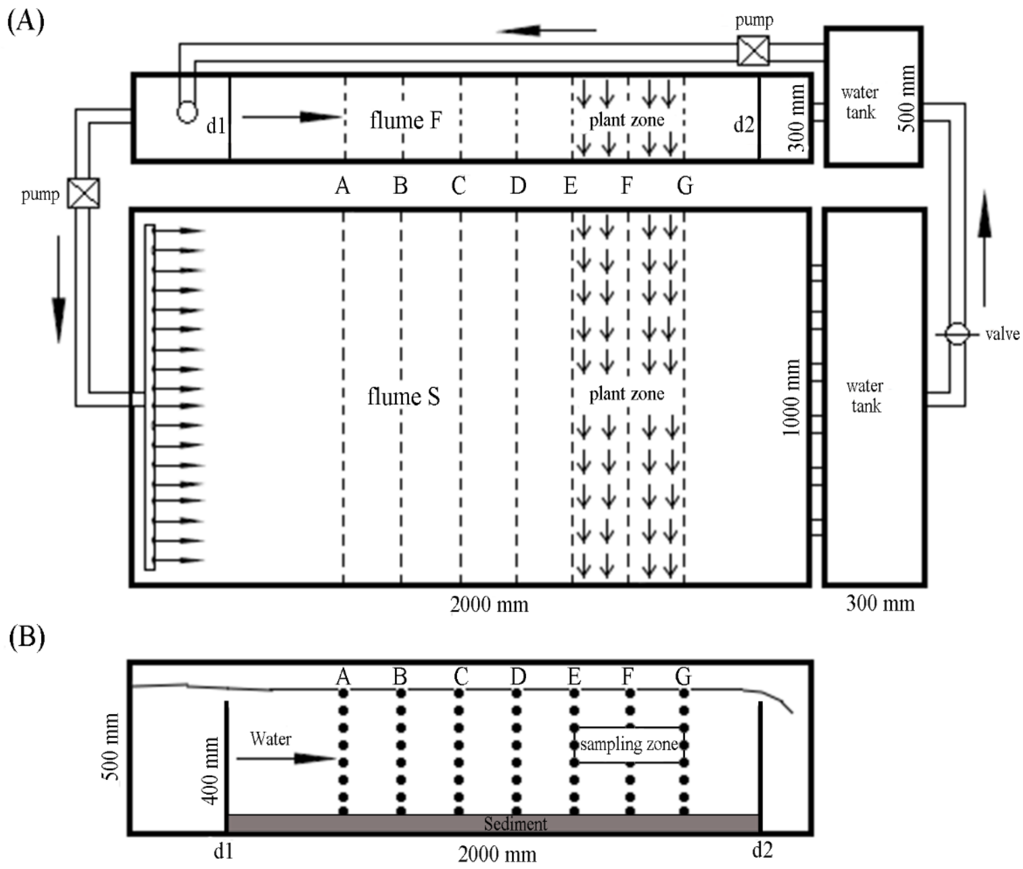


**Supplementary Fig. 1** Experiment facility (**A**) and the measuring points of the current velocities set in the flume F (**B**). The size of the flume F (flow channel) was 2000 × 300 × 500 mm, and the size of the flume S (static tank) was 2000 × 1000 × 500 mm. Water in flume F was circulated and regulated by a water pump and a regulating water tank, whereas water in flume S was circulated and controlled by a water pump, a higher water tank and a valve. To acquire a typical flow condition of open channels in flume F, we added two downflow dams (d1 and d2) near the both ends and their spacing was 1500 mm. $i_F$=1/400, $i_S$=0.

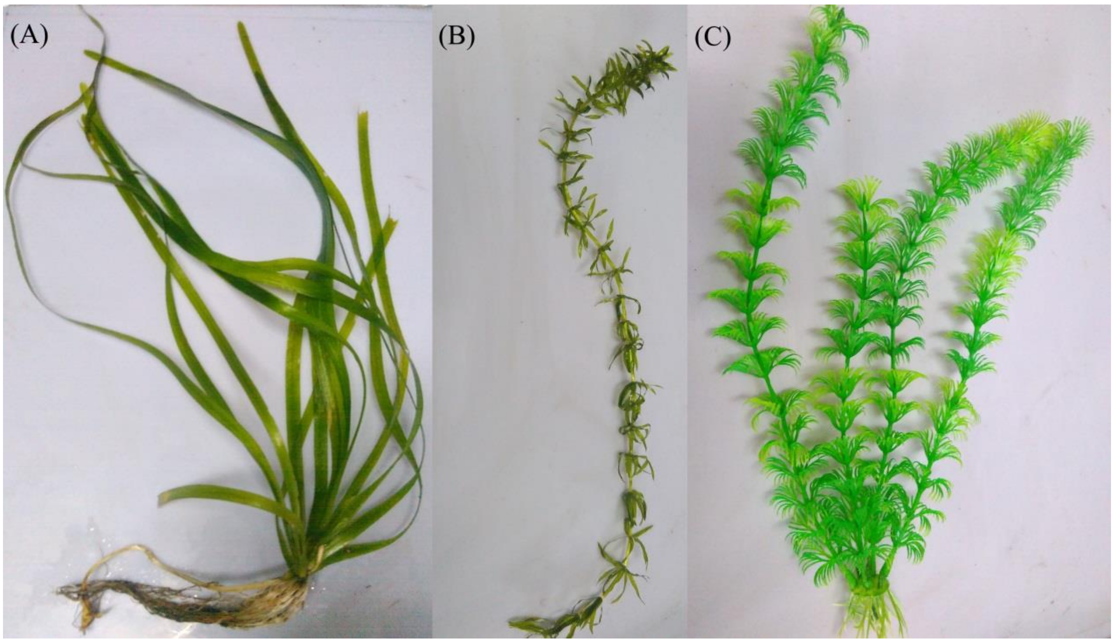


**Supplementary Fig. 2** Plants used in the experiments. (**A**), *Vallisneria natans*; (**B**), *Hydrilla verticillata*; (**C**), artificial plant.

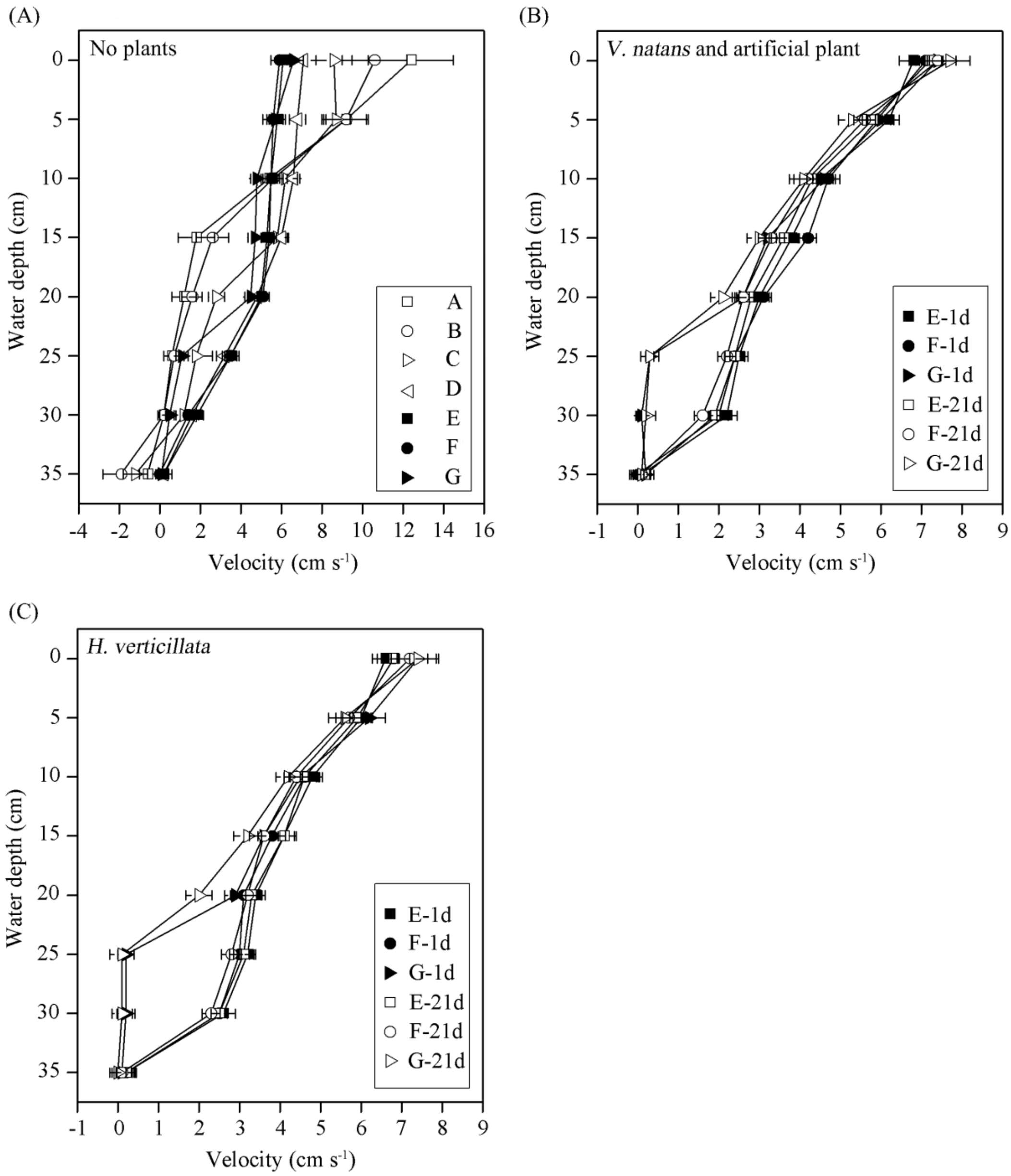


**Supplementary Fig. 3** Distribution of the velocity from cross section A to G without plants (A, *n* = 3), distribution of the velocity from cross section E to G with *V. natans* and artificial plants (B, *n* = 3) and distribution of the velocity from cross section E to G with *H. verticillata* (C, *n* = 3) in the flume F.

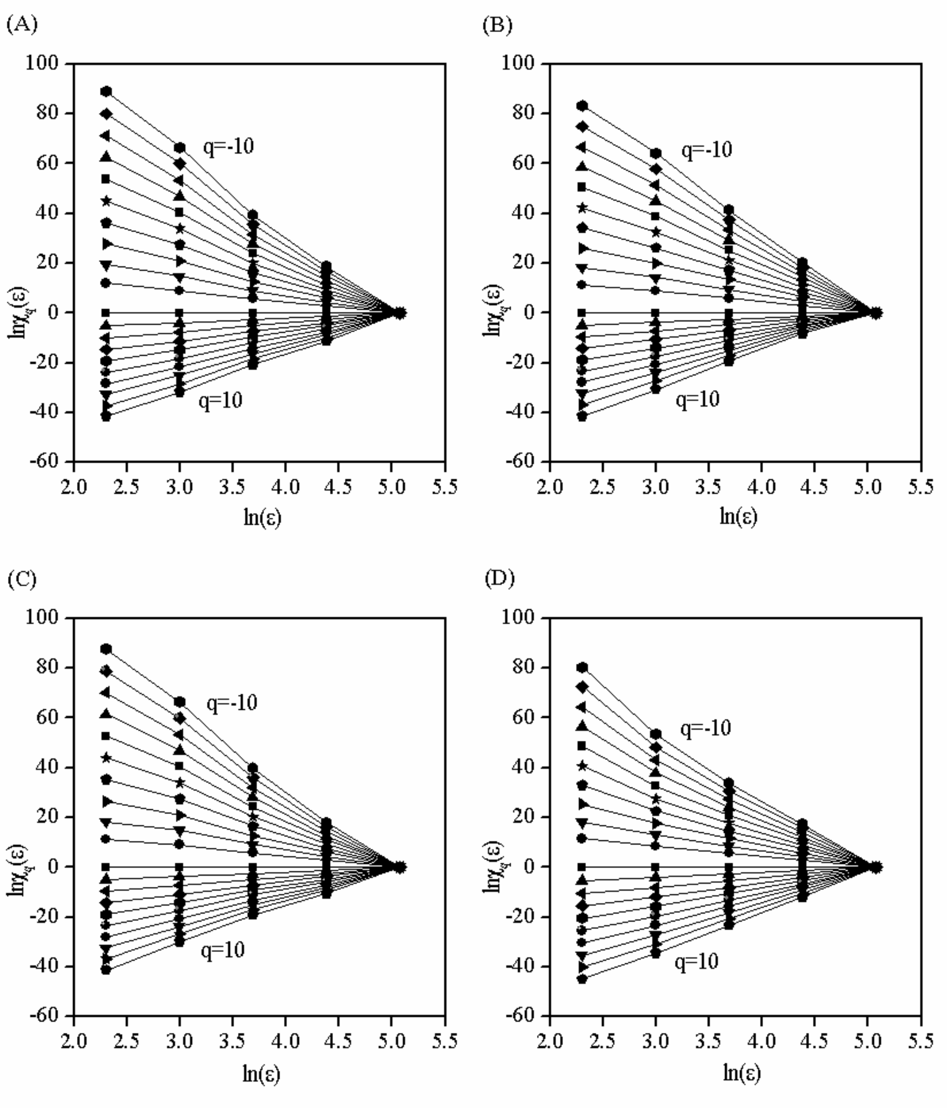


**Supplementary Fig. 4** The graphs of ln $\chi_q(\varepsilon)$-ln ε of the biofilm attached to leaves of two species of plants. (**A**), samples from *V. natans* in static water ($R^2 \geq 0.9957$); (**B**), samples from *V. natans* in flowing water ($R^2 \geq 0.9967$); (**C**), samples from *H. verticillata* in static water ($R^2 \geq 0.9962$); (**D**), samples from *H. verticillata* in flowing water ($R^2 \geq 0.9893$).

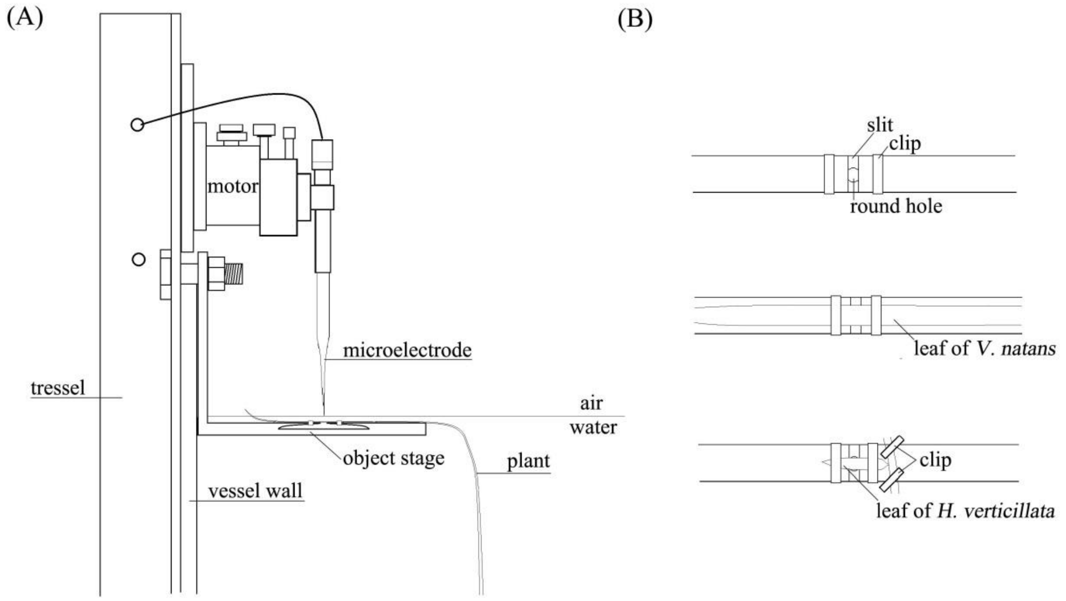


**Supplementary Fig. 5** The *in situ* puncture test with a microelectrode system. (**A**), the facility; (**B**), the structure of the object stage and the way to fix the leaves.

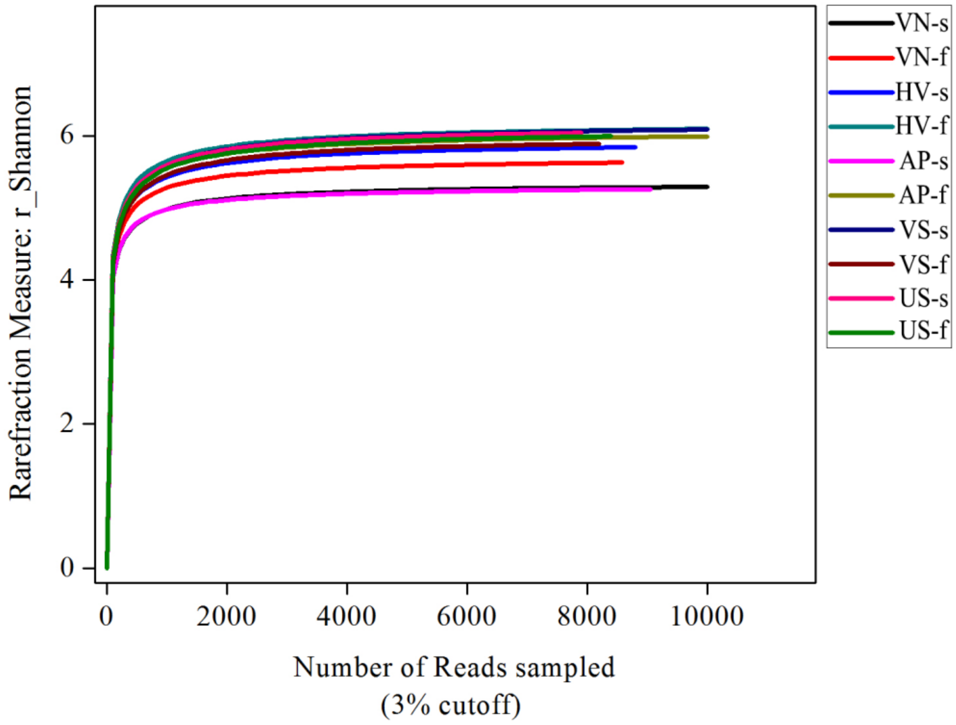


**Supplementary Fig. 6** Rarefaction curves of the 10 samples at cutoff level 3%. VN: *V. natans*; HV: *H. verticillata*; AP: artificial plant; -s: in static water; -f: in flowing water; VS: vegetated sediment; US: unvegetated sediment.

**Supplementary Table 1** Important parameters of multifractal spectra of structure characteristics of biofilms (mean ± s.d., $n$ = 4)

| Plant species | Water condition | $\alpha(q)_{min}$ | $\alpha(q)_{max}$ | $\Delta\alpha = \alpha(q)_{max} - \alpha(q)_{min}$ | $f[\alpha(q)_{min}]$ | $f[\alpha(q)_{max}]$ | $\Delta f = f[\alpha(q)_{min}] - f[\alpha(q)_{max}]$ |
|---|---|---|---|---|---|---|---|
| *V. natans* | static | 1.58±0.06 | 3.18±0.07 | 1.60±0.028 | 0.84±0.055 | 0.27±0.045 | 0.56±0.018 |
| | flowing | 1.61±0.047 | 2.96±0.062 | 1.35±0.019 | 1.23±0.072 | 0.43±0.037 | 0.80±0.039 |
| *H. verticillata* | static | 1.58±0.066 | 2.91±0.056 | 1.33±0.018 | 0.94±0.042 | 0.50±0.03 | 0.44±0.21 |
| | flowing | 1.74±0.05 | 2.88±0.066 | 1.14±0.029 | 1.24±0.036 | 0.23±0.025 | 1.01±0.018 |

**Supplementary Table 2** Raw, clean, normalized, archaeal and bacterial reads, plus numbers of OTUs and Shannon of the samples

| Plant species | Water condition | Reads | | | | | 3% cutoff | |
|---|---|---|---|---|---|---|---|---|
| | | Raw | clean | Normalized | Archaeal | Bacterial* | OTUs | Shannon |
| *V. natans* | static | 15934 | 12152 | 7861 | 48 | 7813 | 754 | 5.2778 |
| | flowing | 14296 | 10045 | 7861 | 134 | 7727 | 856 | 5.6208 |
| *H. verticillata* | static | 14815 | 10666 | 7861 | 195 | 7666 | 1034 | 5.8305 |
| | flowing | 14879 | 11500 | 7861 | 238 | 7623 | 1081 | 6.0723 |
| artificial plant | static | 14093 | 10278 | 7861 | 55 | 7806 | 708 | 5.2502 |
| | flowing | 15960 | 12441 | 7861 | 450 | 7411 | 1059 | 5.97 |
| vegetated sediment | static | 15807 | 14578 | 7861 | 1955 | 5906 | 1163 | 6.0625 |
| | flowing | 10426 | 9254 | 7861 | 2522 | 5339 | 1131 | 5.8835 |
| unvegetated sediment | static | 10318 | 8918 | 7861 | 2148 | 5713 | 1257 | 6.0385 |
| | flowing | 10459 | 9598 | 7861 | 2456 | 5405 | 1106 | 5.9861 |

**Supplementary Table 3** Analysis of the phyla or classes from all the samples according to the detection and comparison of the abundances in different current velocities. The abundance is presented in terms of percentages of the total sequences in a sample. Numbers not less than 0.1% were shown in the table, and numbers larger than 1% were marked in **Bold**.

| Phylum | Class | *V. natans* | | *H. verticillata* | | artificial plant | | vegetated sediment | | unvegetated sedimen | |
|---|---|---|---|---|---|---|---|---|---|---|---|
| | | static | flowing | static | flowing | static | flowing | static | flowing | static | flowing |
| *Euryarchaeota* | *Halobacteria* | / | / | / | / | / | / | **2.29%** | **1.36%** | **1.78%** | **1.46%** |
| | *Methanobacteria* | / | 0.29% | 0.14% | 0.32% | / | 0.18% | 0.43% | 0.56% | 0.61% | 0.45% |
| | *Methanomicrobia* | 0.55% | **1.41%** | **2.33%** | **2.67%** | 0.61% | 5.39% | **14.51%** | **13.41%** | **12.80%** | **17.61%** |
| | *Thermoplasmata* | / | / | / | / | / | / | **2.56%** | **3.54%** | **3.66%** | **3.22%** |
| | *Euryarchaeota_unclassified* | / | / | / | / | / | / | / | / | / | 0.10% |
| | Total | 0.55% | **1.70%** | **2.47%** | **2.99%** | 0.61% | **5.57%** | **19.79%** | **18.87%** | **18.85%** | **22.83%** |
| *Thaumarchaeota* | *AK59* | / | / | / | / | / | / | / | / | / | 0.11% |
| | *AK8* | / | / | / | / | / | / | 0.14% | 0.33% | 0.13% | 0.11% |
| | *Group_C3* | / | / | / | / | / | / | 0.99% | **1.40%** | **1.45%** | **1.22%** |
| | *Marine_Benthic_Group_A* | / | / | / | / | / | / | / | 0.10% | / | / |
| | *Marine_Benthic_Group_B* | / | / | / | / | / | / | 0.37% | 0.79% | 0.51% | 0.62% |
| | *Miscellaneous_Crenarchaeotic_Gr* | / | / | / | / | / | / | **3.09%** | **9.71%** | **5.43%** | **5.71%** |
| | *pSL12* | / | / | / | / | / | / | / | 0.10% | / | / |
| | *Thaumarchaeota_unclassified* | / | / | / | / | / | / | 0.13% | 0.38% | 0.45% | 0.41% |
| | Total | / | / | / | / | / | / | **4.72%** | **12.81%** | **7.96%** | **8.19%** |
| *Crenarchaeota* | *Thermoprotei* | / | / | / | / | / | / | / | / | 0.11% | / |
| *Archaea_norank* | *Archaea_norank_norank* | / | / | / | / | / | / | / | 0.22% | 0.11% | 0.13% |
| *Actinobacteria* | *Acidimicrobiia* | **2.26%** | **1.50%** | 0.52% | 0.62% | **1.18%** | **1.02%** | 0.27% | 0.22% | 0.50% | 0.23% |
| | *Actinobacteria* | **1.74%** | **1.37%** | 0.92% | **1.27%** | **2.05%** | **2.99%** | **1.00%** | 0.70% | **1.21%** | 0.99% |
| | Total | **4.01%** | **2.87%** | **1.44%** | **1.90%** | **3.23%** | **4.01%** | **1.27%** | 0.92% | **1.70%** | **1.22%** |
| | *Bacteroidia* | / | / | 0.39% | / | / | / | 4.33% | 0.14% | / | / |
| | *BD2-2* | / | / | / | / | / | / | **1.22%** | **1.17%** | 0.67% | 0.95% |
| | *BSV13* | / | / | / | / | / | / | 0.37% | 0.32% | 0.34% | 0.18% |
| | *Cytophagia* | 0.22% | **1.22%** | **1.68%** | **2.32%** | 0.51% | 1.58% | 0.53% | 0.34% | 0.29% | 0.25% |

| | | | | | | | | | | | |
|---|---|---|---|---|---|---|---|---|---|---|---|
| *Bacteroidetes* | *Flavobacteriia* | / | 0.11% | 0.23% | 0.60% | 0.42% | 0.20% | 0.37% | 0.27% | 0.42% | 0.46% |
| | *SB-1* | / | / | 0.13% | 0.11% | / | 0.15% | **2.72%** | **2.34%** | **2.58%** | **3.04%** |
| | *SB-5* | / | / | / | / | / | / | 0.90% | 0.61% | 0.37% | 0.83% |
| | *Sphingobacteriia* | **2.48%** | **3.07%** | **4.87%** | **5.83%** | **3.24%** | **3.09%** | **3.08%** | **1.98%** | **3.68%** | **2.87%** |
| | *vadinHA17* | / | / | / | / | / | 0.17% | **3.08%** | **3.43%** | **2.91%** | **3.09%** |
| | *WCHB1-32* | / | / | / | / | / | / | 0.52% | / | / | / |
| | *Bacteroidetes_unclassified* | / | / | / | / | / | / | 0.61% | 0.23% | / | 0.11% |
| | Total | **2.70%** | **4.40%** | **7.30%** | **8.85%** | **4.17%** | **5.19%** | **17.73%** | **10.84%** | **11.27%** | **11.79%** |
| *Candidate_division_WS3* | *Unknown_Class* | / | / | / | / | / | / | / | 0.10% | / | / |
| | *Candidate_division_WS3_norank* | / | / | / | / | / | / | 0.19% | 0.24% | 0.24% | 0.62% |
| | Total | / | / | / | / | / | / | 0.24% | 0.34% | 0.31% | 0.64% |
| *Chlorobi* | *Chlorobia* | 0.14% | 0.28% | / | 0.10% | 0.18% | 0.20% | **2.09%** | 0.17% | 0.11% | 0.10% |
| | *Ignavibacteria* | / | / | / | 0.15% | / | 0.11% | **2.38%** | **1.50%** | **2.62%** | **1.59%** |
| | Total | 0.14% | 0.28% | 0.00% | 0.25% | 0.18% | 0.32% | **4.47%** | **1.67%** | **2.74%** | **1.69%** |
| *Chloroflexi* | *Anaerolineae* | / | / | 0.13% | / | 0.14% | 0.17% | 0.25% | 0.57% | 0.32% | 0.64% |
| | *Caldilineae* | 0.10% | 0.18% | 0.10% | / | / | / | / | / | / | / |
| | *Chloroflexia* | / | / | 0.11% | 0.25% | / | / | / | / | / | / |
| | *Dehalococcoidia* | / | / | / | / | / | / | 0.73% | **3.07%** | **1.14%** | **1.72%** |
| | *Chloroflexi__uncultured* | / | 0.14% | 0.24% | 0.17% | / | 0.20% | 0.51% | 0.10% | 0.15% | 0.34% |
| | *Chloroflexi_unclassified* | / | / | / | / | / | / | 0.10% | 0.11% | 0.28% | 0.29% |
| | Total | 0.10% | 0.32% | 0.59% | 0.42% | 0.14% | 0.37% | **1.59%** | **3.85%** | **1.90%** | **2.99%** |
| *Firmicutes* | *Bacilli* | **2.12%** | **1.44%** | 0.38% | 0.50% | **8.80%** | **1.81%** | 0.14% | 0.23% | 0.10% | 0.11% |
| | *Clostridia* | 0.94% | 0.95% | **2.81%** | **2.21%** | **0.75%** | **2.93%** | **2.32%** | **1.68%** | **1.97%** | **2.44%** |
| | *Erysipelotrichia* | / | 0.15% | / | / | / | / | / | / | / | / |
| | Total | **3.07%** | **2.54%** | **3.19%** | **2.71%** | **9.55%** | **4.73%** | **2.46%** | **1.91%** | **2.07%** | **2.56%** |
| *Lentisphaerae* | *Oligosphaeria* | / | / | / | / | / | / | 0.11% | 0.14% | 0.14% | / |
| | *WCHB1-41* | / | / | / | / | / | / | 0.32% | 0.28% | 0.33% | 0.41% |
| | Total | / | / | / | / | / | / | 0.43% | 0.42% | 0.47% | 0.41% |
| *Planctomycetes* | *OM190* | 0.62% | 0.92% | 0.17% | 0.31% | 0.50% | 0.64% | / | / | / | / |
| | *Phycisphaerae* | 0.50% | 0.73% | 0.33% | 0.70% | 0.33% | 0.89% | 0.32% | 0.76% | 0.47% | 0.67% |
| | *Pla3_lineage* | / | / | / | / | / | / | / | / | 0.13% | / |
| | *Pla4_lineage* | / | / | / | / | / | / | / | 0.11% | 0.14% | 0.25% |
| | *Planctomycetacia* | **2.67%** | **1.81%** | 0.64% | 0.42% | 0.95% | 0.43% | 0.10% | / | / | / |
| | *vadinHA49* | / | 0.11% | / | 0.10% | 0.13% | 0.19% | / | 0.24% | 0.15% | 0.10% |

| | | | | | | | | | | | |
|---|---|---|---|---|---|---|---|---|---|---|---|
| | *Planctomycetes_unclassified* | 0.18% | / | / | / | / | 0.11% | 0.18% | 0.15% | 0.31% | 0.20% |
| | Total | **3.97%** | **3.56%** | **1.13%** | **1.53%** | **1.91%** | **2.26%** | 0.60% | **1.27%** | **1.20%** | **1.23%** |
| *Proteobacteria* | *Alphaproteobacteria* | **24.78%** | **31.06%** | **15.44%** | **18.38%** | **19.35%** | **10.43%** | 0.28% | 0.22% | 0.34% | 0.19% |
| | *Betaproteobacteria* | **41.01%** | **25.63%** | **42.76%** | **32.45%** | **27.39%** | **23.98%** | **12.52%** | **9.80%** | **12.36%** | **10.21%** |
| | *Deltaproteobacteria* | **1.28%** | **3.10%** | **4.61%** | **7.10%** | **1.08%** | **7.95%** | **13.23%** | **14.09%** | **15.61%** | **13.85%** |
| | *Epsilonproteobacteria* | / | / | 0.23% | 0.33% | / | 0.69% | 0.76% | 2.14% | 0.22% | 0.55% |
| | *Gammaproteobacteria* | **7.42%** | **11.45%** | **13.03%** | **12.64%** | **20.51%** | **19.90%** | **3.96%** | **3.68%** | **4.15%** | **3.51%** |
| | *TA18* | / | 0.18% | 0.48% | 0.59% | 0.13% | 0.22% | / | / | / | / |
| | *Proteobacteria_unclassified* | 0.79% | 0.67% | 0.56% | 0.48% | 0.41% | 0.50% | 0.20% | 0.15% | 0.41% | 0.15% |
| | Total | **75.28%** | **72.10%** | **77.10%** | **71.98%** | **68.86%** | **63.66%** | **30.95%** | **30.07%** | **33.09%** | **28.47%** |
| *Verrucomicrobia* | *OPB35_soil_group* | / | 0.20% | / | 0.19% | / | 0.57% | **1.95%** | **1.45%** | **1.82%** | **1.68%** |
| | *Verrucomicrobiae* | 0.59% | **1.13%** | / | / | **2.82%** | 0.38% | / | / | / | / |
| | Total | 0.59% | **1.34%** | 0.00% | 0.19% | **2.82%** | 0.95% | **1.95%** | **1.45%** | **1.82%** | **1.68%** |
| *Acidobacteria* | *Acidobacteria* | 0.37% | **1.64%** | **1.74%** | **1.91%** | 0.93% | **2.14%** | **2.87%** | **3.07%** | **3.47%** | **2.68%** |
| *Armatimonadetes* | *Armatimonadetes_norank* | 0.73% | **1.36%** | 0.32% | 0.46% | 0.25% | 0.50% | / | 0.34% | / | / |
| *BD1-5* | *BD1-5_norank* | / | / | / | / | / | / | 0.18% | 0.29% | 0.28% | 0.15% |
| *Chlamydiae* | *Chlamydiae* | **1.64%** | **2.87%** | **1.08%** | **1.03%** | 0.57% | **3.38%** | 0.42% | 0.65% | 0.50% | 0.39% |
| *Caldiserica* | *Caldisericia* | / | / | / | / | / | / | / | 0.11% | 0.13% | / |
| *Candidate_division_BRC1* | *Candidate_division_BRC1_norank* | / | / | / | / | / | / | / | 0.19% | / | 0.10% |
| *Candidate_division_OD1* | *Candidate_division_OD1_norank* | / | / | / | / | / | / | 0.19% | 0.11% | 0.33% | 0.53% |
| *Candidate_division_OP11* | *Candidate_division_OP11_norank* | / | / | / | / | / | / | / | / | 0.10% | 0.14% |
| *Candidate_division_OP3* | *Candidate_division_OP3_norank* | / | / | / | / | / | / | **2.61%** | **2.96%** | **4.19%** | **3.84%** |
| *Candidate_division_OP8* | *Candidate_division_OP8_norank* | / | / | / | / | / | / | 0.22% | 0.29% | 0.24% | 0.27% |
| *Candidate_division_TM7* | *Candidate_division_TM7_norank* | / | / | / | / | 3.03% | 2.29% | / | / | / | 0.15% |
| *Candidate_division_WS6* | *Candidate_division_WS6_norank* | / | / | / | / | / | / | / | / | 0.15% | / |
| *Cyanobacteria* | *Cyanobacteria* | **4.31%** | **1.39%** | 0.15% | **2.19%** | **1.60%** | 0.62% | / | / | / | / |
| *Deinococcus-Thermus* | *Deinococci* | 0.25% | 0.57% | **1.27%** | 0.65% | 0.32% | 0.19% | / | / | / | 0.17% |
| *Deferribacteres* | *Deferribacteres* | / | / | / | / | / | / | 0.48% | 0.78% | 0.67% | 0.53% |
| *Elusimicrobia* | *Elusimicrobia* | / | / | / | / | / | / | 0.29% | 0.31% | 0.50% | / |
| *Fibrobacteres* | *Fibrobacteria* | / | / | / | / | / | / | 0.38% | / | 0.23% | 0.48% |
| *Gemmatimonadetes* | *Gemmatimonadetes* | / | 0.27% | 0.22% | 0.50% | / | 0.14% | 0.37% | 0.11% | 0.32% | 0.23% |
| *Nitrospirae* | *Nitrospira* | / | 0.22% | / | 0.52% | / | 0.52% | **1.04%** | **2.24%** | **1.00%** | **2.24%** |
| *Spirochaetae* | *Spirochaetes* | / | / | / | / | / | 0.22% | **1.98%** | **1.06%** | **1.44%** | **1.11%** |
| *TA06* | *TA06_norank* | / | / | / | / | / | / | 0.23% | 0.48% | 0.19% | 0.36% |

| | | | | | | | | | | | |
|---|---|---|---|---|---|---|---|---|---|---|---|
| *TM6* | *TM6_norank* | **1.20%** | **1.58%** | 0.79% | 0.64% | **1.13%** | **1.74%** | 0.55% | 0.60% | 0.61% | 0.71% |
| *Bacteria_unclassified* | *Bacteria_unclassified* | 0.39% | 0.48% | 0.36% | 0.51% | 0.18% | 0.28% | 0.81% | 0.97% | **1.11%** | **1.27%** |

**Supplementary Table 4** Parameters of water environment measured every day during the experiment (mean ± s.d., $n$ = 3). DO: dissolved oxygen, Ec: electrical conductivity, ORP: oxidation-reduction potential.

| Species | | *V. natans* and artificial plant | | | | | | | |
|---|---|---|---|---|---|---|---|---|---|
| Water condition | | static | | | | flowing | | | |
| Parameter | | pH | DO (mg L$^{-1}$) | Ec (μS cm$^{-1}$) | ORP (mV) | pH | DO (mg L$^{-1}$) | Ec (μS cm$^{-1}$) | ORP (mV) |
| Day | 1 | 8.12±0.08 | 4.74±0.06 | 310±5.29 | 326.1±3.55 | 8.12±0.06 | 4.73±0.03 | 309.7±1.53 | 325.6±0.72 |
| | 2 | 8.21±0.04 | 4.6±0.03 | 314.7±6.11 | 288.8±5.7 | 8.37±0.03 | 4.58±0.03 | 313±3 | 281±3.36 |
| | 3 | 8.49±0.06 | 5.13±0.04 | 319±4.16 | 280.1±7.56 | 8.51±0.01 | 5.14±0.02 | 317±4.73 | 278.4±1.31 |
| | 4 | 8.51±0.02 | 4.24±0.04 | 321±4.36 | 278.5±6.96 | 8.43±0.03 | 4.22±0.01 | 317±4.36 | 277.6±2.9 |
| | 5 | 8.04±0.02 | 4.25±0.03 | 322±2.65 | 291.1±4.05 | 8.05±0.02 | 4.14±0.02 | 322±3.21 | 277.6±2.9 |
| | 6 | 8.56±0.05 | 4.09±0.02 | 327±2.52 | 244.6±2.27 | 8.58±0.04 | 4±0.04 | 324±1.53 | 243.5±2.48 |
| | 7 | 8.23±0.04 | 4.5±0.03 | 333±1.53 | 287.5±1.48 | 8.25±0.05 | 4.59±0.02 | 330±1.53 | 275.7±0.9 |
| | 8 | 8.6±0.02 | 4.87±0.03 | 334±2.52 | 246.5±6.41 | 8.56±0.05 | 4.84±0.03 | 336±0.58 | 245.3±4.12 |
| | 9 | 8.51±0.01 | 4.87±0.02 | 332±2.08 | 248.3±4.55 | 8.45±0.02 | 4.86±0.02 | 334±1.53 | 247.2±3.2 |
| | 10 | 8.45±0.03 | 4.98±0.03 | 333±2.31 | 250.8±0.91 | 8.5±0.02 | 4.94±0.03 | 334±3.06 | 248.9±2.41 |
| | 11 | 8.54±0.03 | 4.48±0.04 | 340±1.53 | 243.2±1.72 | 8.52±0.02 | 4.55±0.06 | 342±1.53 | 243.9±2.11 |

| | | | | | | | | | |
|---|---|---|---|---|---|---|---|---|---|
| Day | 12 | 8.32±0.03 | 5.04±0.02 | 335±1.53 | 246.1±0.45 | 8.3±0.04 | 4.98±0.02 | 334±2.52 | 242±1.99 |
| | 13 | 8.47±0.04 | 4.47±0.05 | 341±3.51 | 243.9±2.11 | 8.46±0.02 | 4.47±0.04 | 342±0.58 | 243.7±1.37 |
| | 14 | 8.24±0.03 | 4.48±0.07 | 333±2.65 | 281±1.66 | 8.25±0.03 | 4.51±0.03 | 330±0.58 | 279.3±1.6 |
| | 15 | 8.47±0.03 | 5±0.04 | 334±2.08 | 251.7±0.31 | 8.46±0.06 | 4.95±0.03 | 337±1.53 | 249.6±1.35 |
| | 16 | 8.48±0.02 | 4.71±0.03 | 337±2.65 | 247.5±0.95 | 8.49±0.02 | 4.75±0.04 | 335±1.53 | 248.7±3.45 |
| | 17 | 8.57±0.04 | 4.05±0.02 | 332±1.53 | 244.1±1.84 | 8.58±0.02 | 4.01±0.03 | 331±0.58 | 243.9±1.65 |
| | 18 | 8.43±0.02 | 4.53±0.04 | 328±3.61 | 250.1±0.55 | 8.45±0.02 | 4.51±0.01 | 327±3.06 | 247±1.81 |
| | 19 | 8.38±0.02 | 4.39±0.04 | 326±2.65 | 250.4±0.85 | 8.38±0.02 | 4.37±0.02 | 324±2.08 | 251±0.59 |
| | 20 | 8.44±0.03 | 4.29±0.03 | 324±1.53 | 247.8±0.38 | 8.44±0.04 | 4.25±0.02 | 328±1.53 | 247.9±0.4 |
| | 21 | 8.53±0.03 | 4.49±0.02 | 340±1.53 | 247±1.17 | 8.51±0.02 | 4.51±0.03 | 340±1.53 | 245.1±3.02 |

| Species | | *H. verticillata* | | | | | | | |
|---|---|---|---|---|---|---|---|---|---|
| Water condition | | static | | | | flowing | | | |
| Parameter | | pH | DO (mg $L^{-1}$) | Ec (μS $cm^{-1}$) | ORP (mV) | pH | DO (mg $L^{-1}$) | Ec (μS $cm^{-1}$) | ORP (mV) |
| Day | 1 | 8.46±0.07 | 4.98±0.08 | 344.3±7.09 | 277±5.16 | 8.48±0.04 | 5.01±0.02 | 341.7±3.51 | 277.2±3.65 |
| | 2 | 8.27±0.06 | 4.86±0.05 | 339±4.16 | 287±4.23 | 8.25±0.03 | 4.8±0.04 | 347±4.04 | 284.8±3.55 |
| | 3 | 8.53±0.07 | 4.98±0.11 | 342±4.58 | 276±7.01 | 8.53±0.04 | 4.81±0.03 | 331±2.65 | 279.2±2.15 |

| | | | | | | | | | |
|---|---|---|---|---|---|---|---|---|---|
| | 4 | 8.69±0.07 | 5.2±0.06 | 351±4.58 | 268.8±4.05 | 8.75±0.06 | 5.16±0.03 | 353±1.53 | 269.3±2.02 |
| | 5 | 8.5±0.03 | 4.86±0.04 | 351±2.52 | 278.2±2.06 | 8.46±0.03 | 4.84±0.02 | 353±1.15 | 269.7±1.67 |
| | 6 | 8.52±0.03 | 4.97±0.04 | 354±2.08 | 264.4±1.94 | 8.5±0.02 | 4.82±0.01 | 356±1.53 | 264.8±2.1 |
| | 7 | 8.4±0.02 | 5.2±0.02 | 360±2.08 | 269.5±2.41 | 8.36±0.02 | 5.01±0.02 | 359±1.15 | 268±2.26 |
| | 8 | 8.5±0.02 | 4.92±0.03 | 374±2.08 | 262.8±3.39 | 8.48±0.02 | 4.93±0.02 | 374±2.52 | 266.8±1.49 |
| | 9 | 8.32±0.03 | 5±0.06 | 368±1.53 | 260.7±4.04 | 8.3±0.01 | 4.93±0.02 | 372±0.58 | 260.3±1.57 |
| | 10 | 8.31±0.02 | 5.01±0.05 | 369±1.15 | 249.6±1.45 | 8.35±0.02 | 4.98±0.01 | 365±1.53 | 250.4±0.35 |
| | 11 | 8.3±0.01 | 4.77±0.04 | 372±2.08 | 250.8±1.7 | 8.24±0.03 | 4.75±0.05 | 371±1.53 | 251±1.5 |
| Day | 12 | 8.34±0.03 | 4.93±0.02 | 365±1.53 | 249.6±1.12 | 8.37±0.03 | 4.96±0.02 | 366±2.08 | 252.7±1.27 |
| | 13 | 8.32±0.02 | 4.7±0.02 | 368±1.53 | 249.5±0.6 | 8.3±0.02 | 4.7±0.02 | 368±2.08 | 249.5±0.21 |
| | 14 | 8.42±0.02 | 5.02±0.03 | 354±2.52 | 267.1±0.65 | 8.38±0.02 | 5.04±0.02 | 356±1.53 | 268.1±1.23 |
| | 15 | 8.42±0.03 | 5.05±0.07 | 370±2.08 | 249.2±2.21 | 8.38±0.03 | 5.01±0.04 | 368±3 | 250±0.25 |
| | 16 | 8.33±0.03 | 5.02±0.04 | 369±3.21 | 248.7±1.61 | 8.34±0.01 | 5.02±0.02 | 367±1.53 | 250±0.82 |
| | 17 | 8.55±0.02 | 4.74±0.02 | 356±2.08 | 246.4±1.94 | 8.55±0.03 | 4.77±0.04 | 356±1.53 | 247.3±1.16 |
| | 18 | 8.37±0.04 | 5.09±0.03 | 358±1.53 | 269.2±0.85 | 8.42±0.03 | 5.02±0.03 | 357±2.08 | 266.7±1 |
| | 19 | 8.51±0.03 | 5.01±0.03 | 350±2.65 | 265.5±0.89 | 8.51±0.02 | 5.02±0.03 | 352±2.08 | 267.4±2.29 |
| | 20 | 8.39±0.02 | 5.09±0.03 | 348±1.15 | 257.1±0.95 | 8.38±0.02 | 5.07±0.03 | 345±0.58 | 258.3±1.77 |
| | 21 | 8.45±0.03 | 4.64±0.02 | 360±1.53 | 256.6±0.6 | 8.42±0.02 | 4.63±0.02 | 361±0.58 | 254±1.8 |
